# Effects of Shexiang Baoxin Pill for Coronary Microvascular Function: A Systematic Review and Meta-Analysis

**DOI:** 10.3389/fphar.2021.751050

**Published:** 2021-11-02

**Authors:** Mengxi Wang, Yiwen Shan, Weixin Sun, Jie Han, Huaqin Tong, Manlu Fan, Jiandong Chen, Peng Yu, Le Shen, Xiaohu Chen

**Affiliations:** ^1^ Department of Cardiology, Affiliated Hospital of Nanjing University of Chinese Medicine, Nanjing, China; ^2^ Department of Cardiology, Jiangsu Province Hospital of Chinese Medicine, Nanjing, China; ^3^ First Clinical Medical College, Nanjing University of Chinese Medicine, Nanjing, China; ^4^ Department of Cardiology, Yancheng TCM Hospital Affiliated to Nanjing University of Chinese Medicine, Yancheng, China

**Keywords:** shexiang baoxin pil, coronary microvascular function, effect, systematic review, meta-analysis

## Abstract

**Background:** The coronary microvascular dysfunction has attracted more and more attention in recent years, but there is still a lack of effective treatment. Shexiang Baoxin Pill is one of the commonly used drugs for the treatment of coronary artery disease in China. More recently, some studies found that it has the effect of improving coronary microvascular function.

**Objective:** To evaluate the effects of Shexiang Baoxin Pill for coronary microvascular function.

**Methods:** Databases including MEDLINE, Web of Science, CNKI, Wanfang, The Cochrane Library, EMbase, VIP and CBM were searched from inception to June 2021 to screen out relevant clinical studies. The 2019 version 2 of the Cochrane risk of bias tool (RoB2) were used to assess the methodological quality of the included studies. RevMan 5.3 software was used for meta-analysis.

**Results:** Eleven studies meeting the criteria were included, with a total of 1,075 patients. The results of meta-analysis showed that compared with conventional treatment alone, combination of Shexiang Baoxin Pill and conventional treatment can further increase the coronary flow reserve (CFR) [mean difference (MD) = 0.43, 95%CI (0.28, 0.58), *p* < 0.000 01], decrease the index of microvascular resistance (IMR) [MD = −4.23, 95%CI (−5.49, −2.97), *p* < 0.000 01], increase serum nitric oxide (NO) [MD = 11.96, 95%CI (2.74, 21.18), *p* = 0.001] and decrease serum hypersensitive C-reactive protein (hs-CRP) [MD = −2.49, 95%CI (−3.08, −1.90), *p* < 0.000 01], but did not increase the time of duration on the exercise testing (TET) [MD = 3.64, 95%CI (−1.17, 8.45), *p =* 0.14]. In terms of safety, a total of 10 patients developed adverse reactions in the intervention group and 17 patients developed adverse reactions in the control group.

**Conclusion:** Current evidence suggests that Shexiang Baoxin Pill may be effective in the improvement of coronary microvascular function when used in combination with conventional treatment. However, due to the low quality of the included studies, lack of placebo control and high heterogeneity among different studies, we should take a cautious attitude towards this conclusion. Moreover, the safety of Shexiang Baoxin Pill remains uncertain, more high-quality clinical studies are needed to verify the efficacy and safety of this drug in the future.

**Systematic Review Registration:** [website], identifier [registration number: CRD42021265113].

## Introduction

The coronary artery is one of the main arteries in the body which supplies blood to the heart. Atherosclerosis can cause the stenosis of coronary artery, leading to the occurrence of the symptom such as angina and eventually developing into coronary artery disease (Knuuti et al., 2020). Coronary angiography can clearly show the degree and scope of coronary artery stenosis, which is regarded as the gold standard for the diagnosis of coronary artery disease. However, coronary angiography can only reveal epicardial coronary artery with diameter greater than 0.5 mm, and cannot be applied to coronary microvasculature (Mirshahvalad et al., 2020). Coronary microvasculature includes anterior arterioles (about 0.1–0.5 mm in diameter) and arterioles (<0.1 mm in diameter). They provide more than 80% of the circulation resistance of the entire coronary artery system, and play a crucial role in regulating the balance of myocardial blood demand and supply (Mathew et al., 2020). However, the coronary microvascular diseases have not been paid enough attention because the coronary microvasculature are not visible in all kinds of coronary imaging techniques (Vancheri et al., 2020). In recent years, with the pathophysiology research of coronary artery, coronary microvascular diseases have attracted more and more attention in academia. In 2013, European Society of Cardiology (ESC) emphasized the role of coronary microvasculature for the first time in the guidelines on the management of stable coronary artery disease, and officially named such diseases coronary microvascular dysfunction (CMD) (Montalescot et al., 2013). Then, with the deepening of research, the researchers found that more than 40% of angina cases failed to find markedly narrowed vessel in coronary angiography which is mainly caused by CMD (Bucciarelli-Ducci and Pennell, 2010; Sara et al., 2015). At the same time, related research findings also showed that CMD is associated with an increased risk of cardiovascular adverse events and death, regardless of the presence or absence of obstructive coronary artery disease (OCAD) (van de Hoef et al., 2014; Gdowski et al., 2020). These indicate that CMD is seriously threatening human life and has been a significant clinical issue that needs to be solved.

In recent years, great progress has been made in the treatment of OCAD. The β-blocker, angiotensin converting enzyme inhibitor (ACEI), nitrates, calcium channel blocker (CCB), ranolazine and other drugs have shown obvious efficacy in improving the prognosis or symptoms of OCAD (Rousan et al., 2017). However, these drugs did not have a similar effect in treating CMD. The β-blocker has been effective only in some patients with CMD (Bugiardini et al., 1989). The study results on the effects of ACEI and ranolazine for coronary microvascular function are inconsistent (Buus et al., 2004; Mehta et al., 2011; Pauly et al., 2011; Bairey Merz et al., 2016). Nitrate and CCB did not seem to improve the coronary microvascular function (Cannon et al., 1985; Zhang et al., 2017). Therefore, the treatment of CMD still faces great challenge, more safe and effective treatment measures are waiting to be explored.

Coronary artery disease belongs to the category of “*xiongbi*” in Traditional Chinese Medicine (TCM). According to TCM theories, its core pathogenesis is “*yuxue zuzhi xinmai*” and the treatment principle is to “*huoxue huayu*” (Chen et al., 2005). In recent years, with the deepening of the understanding of coronary artery disease, TCM scholars have found that the TCM pathogenesis of OCAD and CMD are not completely the same. Some scholars believe that “*hanning xuemai*” and “*xinluo chuji*” are the main pathogenesis of CMD. Correspondingly, the treatment should focus on “*fangxiang wentong*” (Su et al., 2021). Shexiang Baoxin Pill is a kind of Chinese patent medicine with the function of “*fangxiang wentong*” which is composed of Moschus (the dried preputial secretion of *Moschus berezovskii, M. sifanicus or M. moschiferus*), Radix Ginseng (*Panax ginseng C.A.Mey.*), Bovis Calculus Artifactus (the dried gall-stone of *Bos taurus domesticus Gmelin*), Cinnamomi Cortex (*Cinnamomum cassia*), Styrax (*Liquidambar orientalis Mill.*), Bufonis Venenum (*Bufo bufo gargarizans*), and Borneolum Syntheticum (*Dryobalanops aromatica C.F.Gaertn.*). It has been recommended for the treatment of coronary artery disease by the “Consensus of TCM Experts on the Diagnosis and Treatment of Coronary Artery Disease in China” (Wang and Chen, 2018). In recent years, some researchers have tried to conduct clinical studies on the effect of Shexiang Baoxin Pill for coronary microvascular function and initially confirmed its effectiveness, which attracted more and more attention of this drug in improving coronary microvascular function. In 2016, Du reviewed the clinical trials on Shexiang Baoxin Pill in the treatment of CMD, CMD combined with cardiomyopathy and CMD combined with OCAD (Du, 2016). He drawn the conclusion that Shexiang Baoxin Pill could not only improve coronary microvascular function, but also improve the cardiac function and clinical symptoms of patients with CMD combined with cardiomyopathy or OCAD. Lu, et al. reviewed the pharmacological mechanism of Shexiang Baoxin Pill in improving CMD, and concluded that the underlining mechanisms might related to improving endothelial cell function and inhibiting inflammatory response (Lu et al., 2016). Although more and more studies have shown that Shexiang Baoxin pill may have the effect of improving coronary microvascular function, the sample size of relevant clinical trials is too small to provide enough evidence. Therefore, we conducted this systematic review and meta-analysis to evaluate the effects of Shexiang Baoxin Pill for coronary microvascular function aiming to provide more evidence for TCM treatment on CMD.

## Materials and Methods

This systematic review and meta-analysis followed the Preferred Reporting Items for Systematic Reviews and Meta-Analyses (PRISMA) statement and has been registered in PROSPERO (registration number: CRD42021265113).

### Database and Search Strategies

We searched the following databases from their inception to June 2021: MEDLINE, Web of Science, China National Knowledge Infrastructure database (CNKI), Wan-fang database (wanfang), The Cochrane Library, embase, Chinese Scientific Journals database (VIP) and Chinese Biomedicine database (CBM). The publishing language was restricted to Chinese and English. The retrieval words including: “Shexiang Baoxin”, “Shexiang Baoxin Pill”, “Shexiang Baoxin wan”, “coronary microvascular”, “coronary artery microvascular”, “coronary microvasculature”, “coronary artery microvasculature”, “coronary microvessel”, “coronary artery microvessel”, “coronary micrangium”, “coronary artery micrangium”, “coronary capillary”, “coronary artery capillary”, “coronary microcirculation”, “coronary artery microcirculation”, “syndrome X”, “X syndrome”, “coronary flow reserve”, “CFR”, “index of microvascular resistance” and “IMR”. We adopted the search strategy of combining subject words and free words. In addition, we also manually retrieved the references of published literature to search additional relevant studies.

### Inclusion Criteria

Studies meeting the following criteria were included: 1) randomized controlled trial to compare the effects of conventional treatment and conventional treatment combined with Shexiang Baoxin Pill on coronary microvascular function; 2) the control group received conventional treatment including aspirin, P2Y12 receptor inhibitors, statins, β-blocker, ACEI, ARB, CCB, nitrates and nicorandil; 3) the intervention group received conventional treatment combined with Shexiang Baoxin Pill (produced by Shanghai Hutchison Pharmaceuticals Company, oral administration, 45 mg at a time, three times a day); 4) reported one of the following outcomes at least, the primary outcomes are as follows, 1) coronary flow reserve (CFR), 2) index of microvascular resistance (IMR); the secondary outcomes include as follows, 1) serum nitric oxide (NO), 2) serum hypersensitive C-reactive protein (hs-CRP), 3) time of duration on the exercise testing (TET).

### Exclusion Criteria

Studies meeting the following criteria were excluded: 1) the data is incomplete; 2) case reports, reviews, conference literature, theoretical discussions and experience summaries; 3) the baseline information of patients were inconsistent.

### Data Extraction

Two researchers independently screened the retrieved literature on the basis of inclusion and exclusion criteria. They extracted the data, checked with each other and solved divergences by discussing with the third researcher. The extracted data content includes first author name, published time, sample size, gender distribution, average age, intervention measures, treatment duration, comorbidity, outcomes and adverse reactions.

### Quality Evaluation

The methodological quality of included literature was evaluated on the basis of the 2019 version 2 of the Cochrane risk of bias tool (RoB2). This risk of bias assessment includes the following five domains: 1) bias arising from the randomization process; 2) bias due to deviations from intended interventions; 3) bias due to missing outcome data; 4) bias in measurement of the outcome; 5) bias in selection of the reported result. Finally, a judgment of overall risk of bias is generated. There are three levels of bias risk: “low”, “high”, and “some concerns”.

### Data Analysis

We used the RevMan 5.3 software to perform the statistical analysis. Relative risk (RR) was employed for effect measure of dichotomous variables and mean difference (MD) was used for effect measure of continuous variables. In addition, 95% confidence interval (CI) was calculated for both variables. Heterogeneity was evaluated by the *χ* ^*2*^ test and the *I* ^*2*^ statistic. If substantial heterogeneity existed (*I* ^*2*^ > 50% or *p* < 0.05), we applied random effect model to data analysis; otherwise, we applied fixed effect model. Meanwhile, subgroup analysis and sensitivity analysis were applied to explore the sources of heterogeneity and verify the stability of the meta-analysis results. Finally, we used funnel plots to evaluate the publication bias.

## Results

### Search Results

**FIGURE 1:**
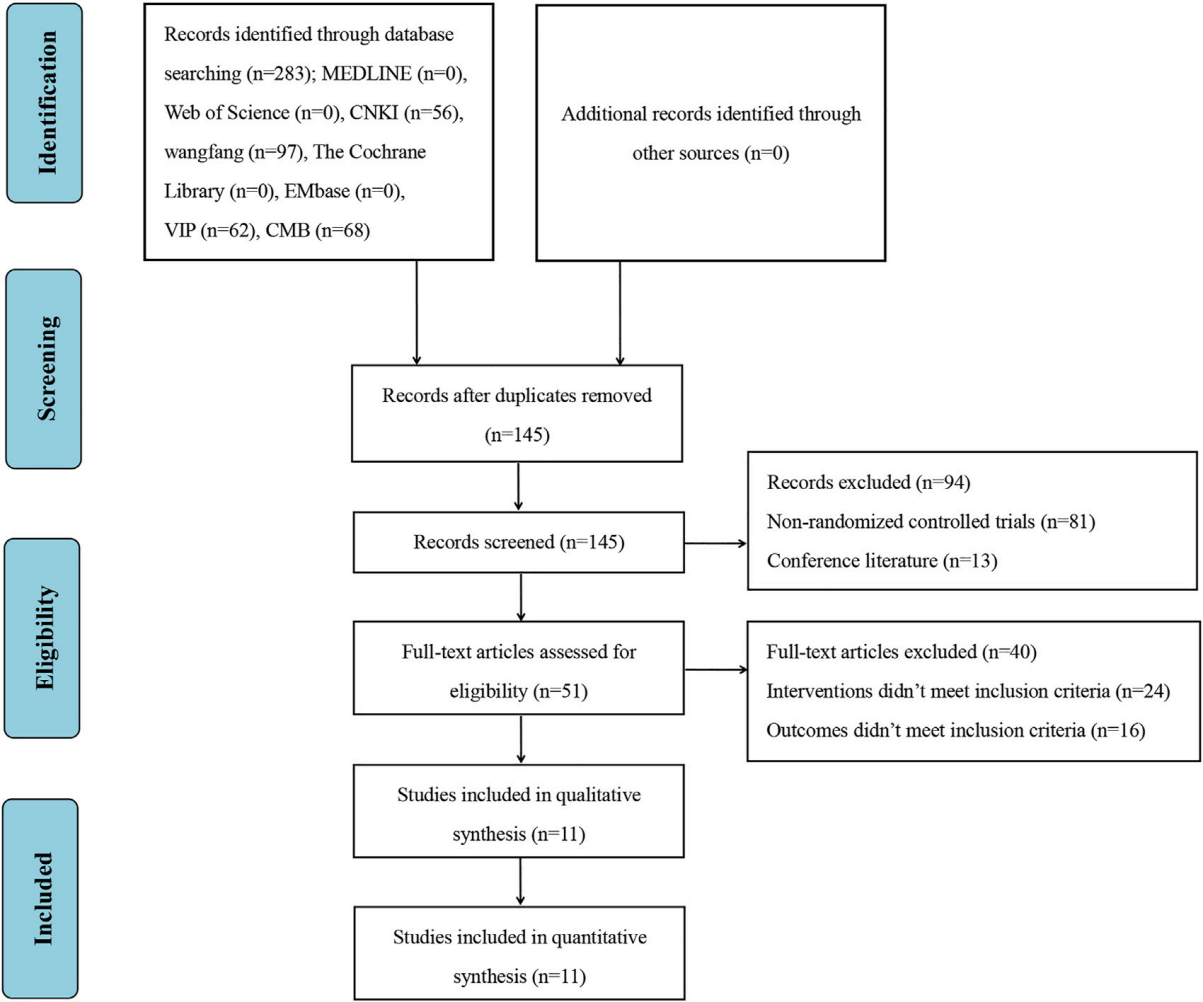
Flow diagram of literature screening.

A total of 283 studies were retrieved from the database. We used the EndNote software to eliminate 138 duplicate studies. After reading the titles and abstracts, we excluded 94 studies in that they were conference literature or non-randomized controlled trials. After reading the full texts, we excluded 40 studies because their interventions measures or outcomes didn’t meet inclusion criteria. Finally, 11 studies were included for the systematic review and meta-analysis (Song et al., 2011; Wang, 2015; Chen et al., 2016; Wu et al., 2019; Zhang, 2019; Sun and Wang, 2020; Zhang et al., 2020; Fu, 2021; Shen et al., 2021; Song et al., 2021; Yan, 2021). The screening process is visualized in Figure 1.

### Study Characteristics

The 11 studies involved a total of 1,075 patients, (552 in intervention group and 443 in control group). The detailed characteristics of these studies are presented in Table 1.

**TABLE 1:** Basic characteristics of included studies.

Study	Sample size	Age (years)	Sex M/F	Intervention measures		Treatment duration (days)	With OCAD	Outcomes	Adverse events
	I/C	I/C	I/C	I	C				
Chen XL 2016	37/33	I: 58.00 ± 13.00	I: 10/27	P + S + B + A + C + N + SBP 45 mg tid	P + S + B + A + C + N	180	not	①②	Not reported
		C: 57.00 ± 15.00	C: 9/24						
Fu CH 2021	82/82	I: 61.48 ± 12.49	I: 52/30	P + S + B + A + C + N + SBP 45 mg tid	P + S + B + A + C + N	56	not	③④	Not reported
		C: 62.13 ± 11.57	C: 54/28						
Shen SX 2021	32/32	I: 41.38 ± 9.43	I: 20/12	P + S + B + A + C + N + SBP 45 mg tid	P + S + B + A + C + N	365	not	②	Not reported
		C: 47.75 ± 10.61	C: 18/14						
Song KY2011	74/68	I: 50.00 ± 7.00	I: 42/32	P + S + B + A + C + N + SBP 45 mg tid	P + S + B + A + C + N	180	not	①	Not reported
		C: 51.00 ± 9.00	C: 39/29						
Song Z2021	50/50	I: 56.40 ± 8.60	I: 30/20	P + S + B + A + C + N + SBP 45 mg tid	P + S + B + A + C + N	90	yes	①②④	Occurrence
		C: 55.90 ± 7.70	C: 35/15						
Sun XY 2020	64/64	I: 72.55 ± 3.63	I: 32/32	P + S + B + A + C + N + SBP 45 mg tid	P + S + B + A + C + N	180	yes	①②③	Not reported
		C: 72.34 ± 3.46	C: 32/32						
Wang HZ2015	20/20	I: 58.00 ± 3.00	I: 8/12	P + S + B + A + C + N + SBP 45 mg tid	P + S + B + A + C + N	56	not	⑤	None
		C: 59.00 ± 4.00	C: 7/13						
Wu CY 2019	38/38	I: 53.70 ± 2.60	I: 24/14	P + S + B + A + C + N + D + SBP 45 mg tid	P + S + B + A + C + N + D	90	not	③④⑤	Occurrence
		C: 54.10 ± 2.50	C: 23/15						
Yan XY 2021	50/41	I: 66.70 ± 6.00	I: 26/24	P + S + B + A + C + N + D + SBP 45 mg tid	P + S + B + A + C + N + D	90	yes	③④	Not reported
		C: 66.10 ± 6.40	C: 21/20						
Zhang HF2020	60/50	I: 48.10 ± 5.12	I: 36/24	P + S + B + A + C + N + SBP 45 mg tid	P + S + B + A + C + N	180	yes	①②④	Not reported
		C: 48.15 ± 5.10	C: 30/20						
Zhang LW2019	45/45	I: 58.62 ± 6.13	I: 24/21	P + S + B + A + C + N + SBP 45 mg tid	P + S + B + A + C + N	84	yes	①②	Not reported
		C: 59.03 ± 6.25	C: 25/20						

I, intervention group; C, control group; M, male; F, female; SBP, Shexiang Baoxin Pill; OCAD, obstructive coronary artery disease; P, antiplatelet drug; S, statins; B, β-blocker; A, ACEI or ARB; C, CCB; N, nitrates; D, nicorandil; ① CFR; ② IMR; ③ NO; ④ hs-CRP; ⑤ TET.

### Quality Assessment of Included Studies

**FIGURE 2:**
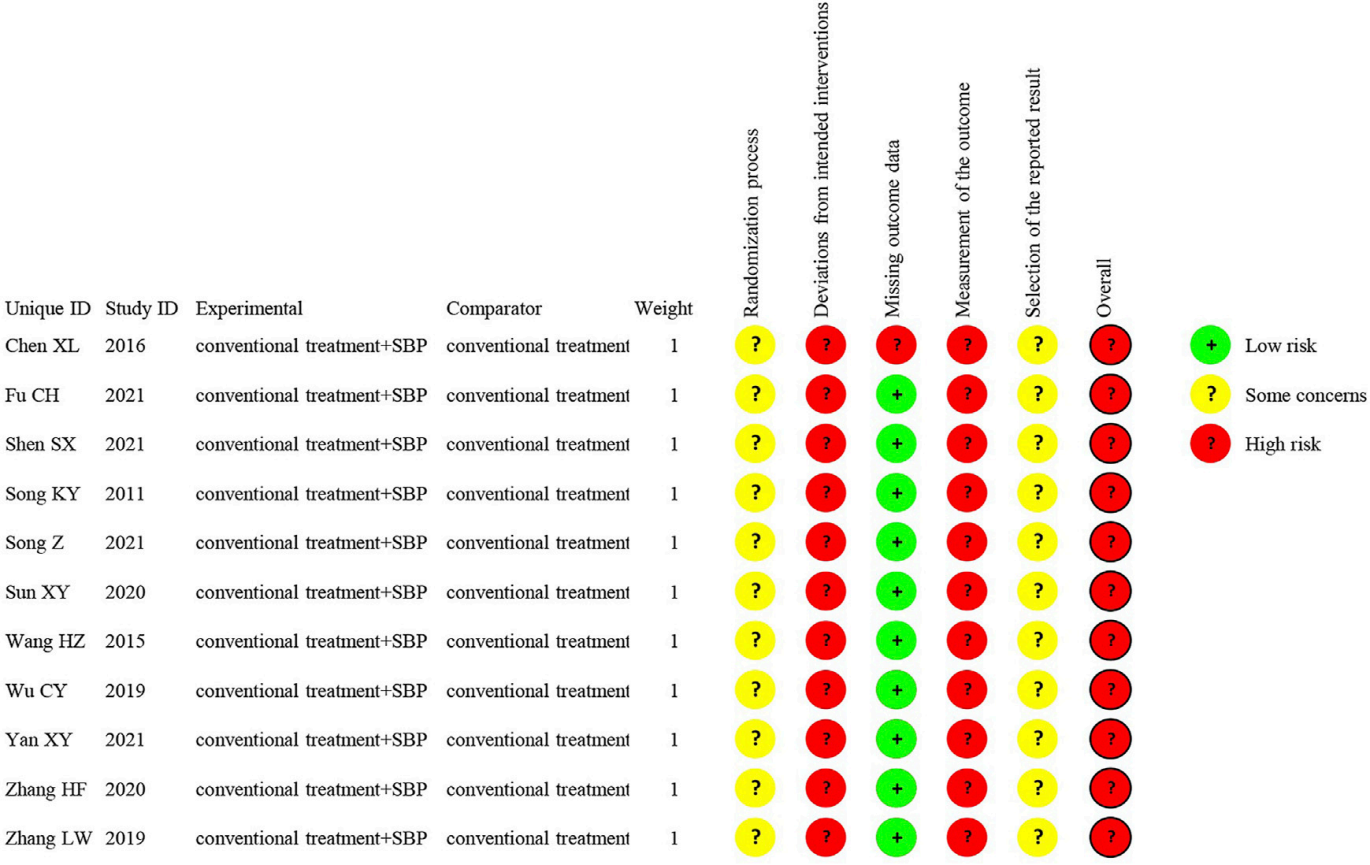
Risk of bias graph.

Four studies (Sun and Wang, 2020; Fu, 2021; Shen et al., 2021; Song et al., 2021) used the random number table method to group patients and seven studies did not report randomization method (Song et al., 2011; Wang, 2015; Chen et al., 2016; Wu et al., 2019; Zhang, 2019; Zhang et al., 2020; Yan, 2021). None of the 11 studies reported allocation concealment method and blinding. One studies reported six patients lost to follow-up but did not elaborate on the reason (Chen et al., 2016). All of the 11 studies reported predetermined outcomes and none reported other biases. The Cochrane bias risk results are presented in Figure 2. A detailed assessment process is provided in Supplementary Table S1 of Bias Risk Evaluation Process.

### Efficacy Assessment

#### Primary Outcomes

##### Coronary Flow Reserve

**FIGURE 3:**
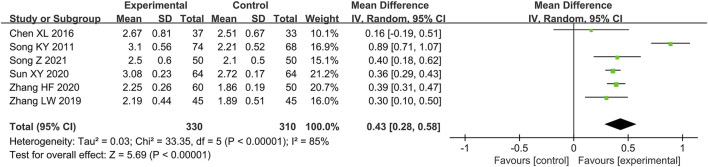
Forest plot of CFR.

Six studies (Song et al., 2011; Chen et al., 2016; Zhang, 2019; Sun and Wang, 2020; Zhang et al., 2020; Song et al., 2021) reported the CFR as outcome. Due to the high heterogeneity (*p* < 0.000 01, *I* ^*2*^ = 85%), we conducted the subgroup analysis to explore the sources of heterogeneity based on treatment duration (more than or not more than 4 months), average age (more than or not more than 55 years old), comorbidity (with OCAD or without OCAD), gender distribution (male account for more than or not more than 50%) and sample size (more than or not more than 100 patients). The results show the heterogeneity was significantly reduced in subgroups of treatment duration not more than 4 months, average age more than 55 years old, comorbid OCAD, male account for not more than 50% and sample size not more than 100 patients (Table 2) (Supplementary Figures S1–S5), indicating that all these factors may be the source of heterogeneity. However, it should be noted that there is still considerable heterogeneity in the remaining subgroups (Table 2) (Supplementary Figures S1–S5), suggesting that heterogeneity may come from other sources. Since the CFR is susceptible to many factors, leading to certain errors in measurement in different research institutions, we consider that heterogeneity might result from this. In addition to the above factors, we found no other significant differences in clinical and methodological features between the six studies. We believed that these clinical differences are acceptable when exploring the efficacy of drugs in the whole population, so we applied random effect model to pool the data of six studies. The result indicated that combination of conventional treatment and Shexiang Baoxin Pill can further increase CFR compared with conventional treatment alone [MD = 0.43, 95%CI (0.28, 0.58), *p* < 0.000 01] (Figure 3).

**TABLE 2:** Subgroup analysis of CFR based on treatment duration, average age, comorbidity, gender distribution and sample size.

Criteria for grouping	Subgroup	n	MD (95%CI)	*I* ^*2*^ (%)	*Z*	*P*
treatment duration	more than 4 months	4	0.47 (0.27, 0.67)	91	4.56	<0.000 01
	less than 4 months	2	0.35 (0.20, 0.49)	0	4.65	<0.000 01
average age	more than 55 years old	4	0.35 (0.29, 0.41)	0	11.07	<0.000 01
	less than 55 years old	2	0.63 (0.14, 1.12)	96	2.54	0.01
Comorbidity	with OCAD	4	0.37 (0.32, 0.42)	0	14.31	<0.000 01
	without OCAD	2	0.54 (-0.17, 1.26)	93	1.48	0.14
gender distribution	male account for more than 50%	4	0.49 (0.25, 0.74)	89	3.97	<0.000 1
	male account for less than 50%	2	0.34 (0.21, 0.46)	18	5.08	<0.000 01
sample size	more than 100 patients	3	0.53 (0.31, 0.75)	93	4.67	<0.000 01
	less than 100 patients	3	0.32 (0.18, 0.45)	0	4.63	<0.000 01

OCAD, obstructive coronary artery disease; n, number of studies.

##### Index of Microvascular Resistance

**FIGURE 4:**
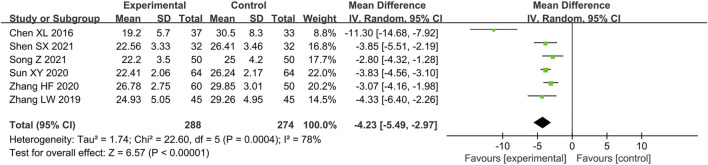
Forest plot of IMR.

Six studies (Chen et al., 2016; Zhang, 2019; Sun and Wang, 2020; Zhang et al., 2020; Shen et al., 2021; Song et al., 2021) reported the IMR as outcome. Due to the high heterogeneity (*p =* 0.000 4, *I* ^*2*^ = 78%), we also conducted subgroup analysis based on the above criteria to explore the source of heterogeneity. The results show the heterogeneity was significantly reduced in subgroups of treatment duration not more than 4 months, average age not more than 55 years old, comorbid OCAD, male account for more than 50% and sample size more than 100 patients (Table 3) (Supplementary Figures S6–S10), indicating that all these factors may be the source of heterogeneity. Similar to the result of CFR, there is still considerable heterogeneity in the remaining subgroups (Table 3) (Supplementary Figures S6–S10), suggesting that heterogeneity may come from other sources. Since the IMR is a newly discovered indicator in recent years, there may be some differences in the level of measurement technology in different hospitals, we speculated that heterogeneity may be related to this. For the similar reason to the CFR, we also applied random effect model to pool the data of six studies. The result indicated that combination of conventional treatment and Shexiang Baoxin Pill can further decrease IMR compared with conventional treatment alone [MD = -4.23, 95%CI (-5.49, -2.97), *p* < 0.000 01] (Figure 4).

**TABLE 3:** Subgroup analysis of IMR based on treatment duration, average age, comorbidity, gender distribution and sample size.

Criteria for grouping	Subgroup	n	MD (95%CI)	*I* ^*2*^ (%)	*Z*	*P*
treatment duration	more than 4 months	4	−4.74 (−6.53, −2.95)	86	5.19	<0.000 01
	less than 4 months	2	−3.40 (−4.86, −1.93)	27	4.55	<0.000 01
average age	more than 55 years old	4	−5.00 (−7.16, −2.84)	85	4.55	<0.000 01
	less than 55 years old	2	−3.30 (−4.21, −2.39)	0	7.12	<0.000 01
Comorbidity	with OCAD	4	−3.54 (−4.09, −3.00)	0	12.76	<0.000 01
	without OCAD	2	−7.42 (−14.72, −0.13)	93	1.99	0.05
gender distribution	male account for more than 50%	4	−3.31 (−4.04, −2.58)	0	8.90	<0.000 01
	male account for less than 50%	2	−7.38 (−14.69, −0.06)	94	1.98	0.05
sample size	more than 100 patients	2	−3.56 (−4.27, −2.85)	23	9.79	<0.000 01
	less than 100 patients	4	−5.18 (−7.79, −2.57)	85	3.89	<0.000 1

OCAD, obstructive coronary artery disease; n, number of studies.

### Secondary Outcomes

#### Nitric Oxide (μmol/L)

**FIGURE 5:**
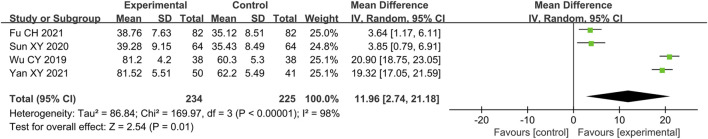
Forest plot of NO.

Four studies (Wu et al., 2019; Sun and Wang, 2020; Fu, 2021; Yan, 2021) reported the NO as outcome. Due to the high heterogeneity (*p* < 0.000 01, *I* ^*2*^ = 98%), we also conducted subgroup analysis based on the above criteria to explore the source of heterogeneity. The results show the heterogeneity was significantly reduced in subgroups of sample size more than 100 patients and sample size not more than 100 patients (Table 4) (Supplementary Figures S11–S15), indicating that sample size may be the source of heterogeneity. For the similar reason to the CFR, we applied random effect model to pool the data of four studies. The result indicated that combination of conventional treatment and Shexiang Baoxin Pill can further increase NO compared with conventional treatment alone [MD = 11.96, 95%CI (2.74, 21.18), *p* = 0.001] (Figure 5).

**TABLE 4:** Subgroup analysis of NO based on treatment duration, average age, comorbidity, gender distribution and sample size.

Criteria for grouping	Subgroup	n	MD (95%CI)	*I* ^*2*^ (%)	*Z*	*P*
treatment duration	more than 4 months	1	3.85 (0.79, 6.91)	—	2.47	0.01
	less than 4 months	3	14.63 (4.23, 25.04)	98%	2.76	0.006
average age	more than 55 years old	3	8.96 (−1.85, 19.77)	98%	1.62	0.10
	less than 55 years old	1	20.90 (18.75, 23.05)	—	19.05	<0.000 01
comorbidity	with OCAD	2	11.62 (-3.54, 26.78)	98%	1.50	0.13
	without OCAD	2	12.28 (-4.63, 29.20)	99%	1.42	0.15
gender distribution	male account for more than 50%	3	14.63 (4.23, 25.04)	98%	2.76	0.006
	male account for less than 50%	1	3.85 (0.79, 6.91)	—	2.47	0.01
sample size	more than 100 patients	2	3.72 (1.80, 5.65)	0%	3.79	0.000 1
	less than 100 patients	2	20.15 (18.59, 21.71)	0%	25.30	<0.000 01

OCAD, obstructive coronary artery disease; n, number of studies.

#### Hypersensitive C-Reactive Protein (mg/L)

**FIGURE 6:**
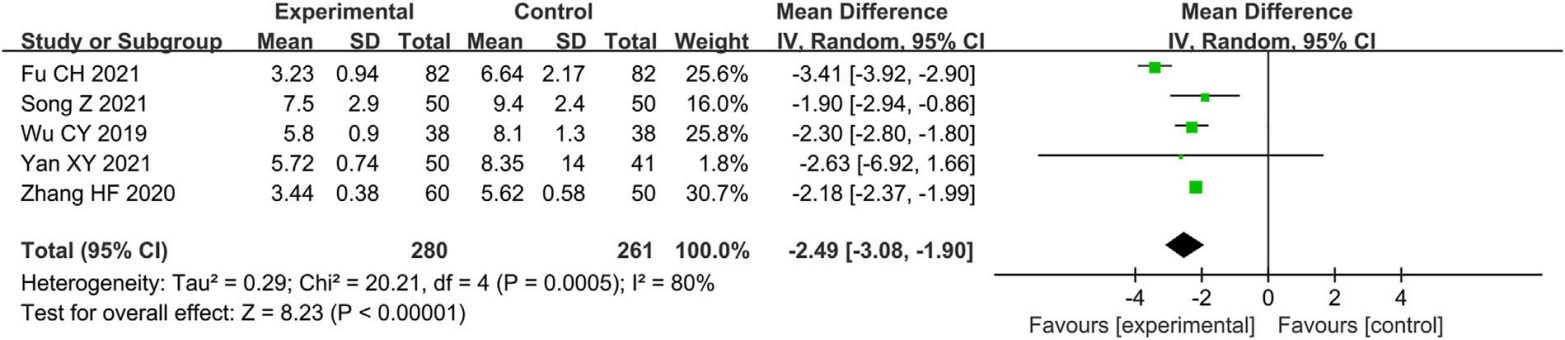
Forest plot of hs-CRP.

Five studies (Wu et al., 2019; Zhang et al., 2020; Fu, 2021; Song et al., 2021; Yan, 2021) reported the hs-CRP as outcome. Due to the high heterogeneity (*p =* 0.000 5, *I* ^*2*^ = 80%), we conducted the subgroup analysis to explore the sources of heterogeneity. Since males account for more than 50% of all five studies, we did not conduct subgroup analysis based on gender distribution. The results show the heterogeneity was significantly reduced in subgroups of average age less than 55 years old, comorbid OCAD and sample size less than 100 patients (Table 5) (Supplementary Figures S16–S19), indicating that average age, comorbidity and sample size may be the source of heterogeneity. In addition, there is still considerable heterogeneity in the remaining subgroups (Table 5) (Supplementary Figures S16–S19), suggesting that heterogeneity may come from other sources. We believed that the differences in detection equipment and techniques used in different studies may be one of the main sources. For the similar reason to the CFR, we applied random effect model to pool the data of five studies. The result indicated that combination of conventional treatment and Shexiang Baoxin Pill can further decrease hs-CRP compared with conventional treatment alone [MD = -2.49, 95%CI (-3.08, -1.90), *p* < 0.000 01] (Figure 6).

**TABLE 5:** Subgroup analysis of hs-CRP based on treatment duration, average age, comorbidity and sample size.

Criteria for grouping	Subgroup	n	MD (95%CI)	*I* ^*2*^ (%)	*Z*	*P*
treatment duration	more than 4 months	1	−2.18 (−2.37, −1.99)	—	22.81	<0.000 01
	less than 4 months	4	−2.61 (−3.45, −1.76)	75%	6.06	<0.000 01
average age	more than 55 years old	3	−2.73 (−4.01, −1.46)	69%	4.21	<0.000 1
	less than 55 years old	2	−2.19 (−2.37, −2.02)	0%	24.50	<0.000 01
Comorbidity	with OCAD	3	−2.17 (−2.36, −1.99)	0%	23.11	<0.000 01
	without OCAD	2	−2.85 (−3.94, −1.77)	89%	5.14	<0.000 01
sample size	more than 100 patients	2	−2.77 (−3.98, −1.57)	95%	4.51	<0.000 01
	less than 100 patients	3	−2.23 (−2.68, −1.78)	0%	9.70	<0.000 01

OCAD, obstructive coronary artery disease; n, number of studies

#### Time of Duration on the Exercise Testing (min)

**FIGURE 7:**
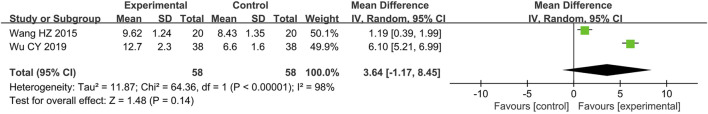
Forest plot of TET.

Two studies (Wang, 2015; Wu et al., 2019) reported the TET as outcome. Although the heterogeneity was high (*p* < 0.000 01, *I* ^*2*^ = 98%), we were unable to conduct a subgroup analysis because there were only two studies. After comparing the detailed characteristics of the two studies, we considered the clinical heterogeneity and methodological heterogeneity can be accepted, so we applied random effect model to pool the data. The result indicated that combination of conventional treatment and Shexiang Baoxin Pill can further increase TET compared with conventional treatment alone, but the difference was not statistically significant [MD = 3.64, 95%CI (-1.17, 8.45), *p =* 0.14] (Figure 7).

### Safety

Only three included studies reported the incidence of adverse reactions (the total sample size was 216, including 108 patients in the control group and 108 patients in the intervention group) (Wang, 2015; Wu et al., 2019; Song et al., 2021). One of the studies found no adverse reactions occurred during follow-up (the sample size was 40, including 20 patients in the control group and 20 patients in the intervention group) (Wang, 2015). Two of the studies reported a total of 10 patients developed adverse reactions in the intervention group (two patients developed tongue numbness, two patients developed alimentary tract reactions, one patients developed palpitation, two patients developed rash, three patient had bleeding events) and 17 patients developed adverse reactions in the control group (two patients developed headache, two patients developed inappetence, one patients developed alimentary tract reactions, three patients developed palpitation, four patients developed rash, five patient had bleeding events) (Wu et al., 2019; Song et al., 2021). It seems that Shexiang Baoxin Pill is safe. However, it should be noted that only three studies with 216 patients in total reported the incidence of adverse reactions, which was too small to provide enough evidence. Some other clinical studies which are not included found that Shexiang Baoxin Pill had adverse reactions such as decreased blood pressure and heart rate, abnormal liver and kidney function during treatment course, which are not reported in our included studies (Wen et al., 2018). In addition, the side effects mentioned in the instructions of Shexiang Baoxin Pill is tongue numbness only, and in the Precautions and Contraindications parts, it only prompts that the athletes should use with caution and pregnant women should avoid. The lack of detailed description of safety and sufficient literature data support makes the safety of this drug uncertain. As we all know, this is one of the biggest obstacles to the worldwide promotion of most Chinese patent medicine. Encouragingly, several high-quality clinical trials in recent years have provided strong evidence for the safety of Shexiang Baoxin Pill (Ge et al., 2021). However, due to the lack of sufficient safety evidence in patients with coronary microvascular disease, we should keep a relatively cautious attitude towards the drug. It is expected that more high-quality clinical trials can be conducted in the future to provide more sufficient evidence for the use of this drug in coronary microvascular diseases.

### Publication Bias

The publication bias assessment was not be conducted because each of the outcomes included less than 10 studies.

## Discussion

Through the analysis of 1,075 patients, we found that compared with conventional treatment alone, combination of Shexiang Baoxin Pill and conventional treatment could further increase CFR and NO, decrease IMR and hs-CRP, but has no obvious effect on TET. In terms of safety, the results showed that no serious adverse reactions occurred in all the included studies, and the incidence of adverse reactions in the intervention group was not higher than control group. It seems that Shexiang Baoxin Pill is safe. However, only three studies reported the occurrence of adverse reactions, which was not enough to provide convincing evidence, so we believe that the safety of this drug is not clear, more studies are needed to confirm it in the future. Furthermore, as can be seen, all the outcomes of our review were surrogate end-point instead of clinical end-point. This is because the clinic trials on the effects of Shexiang Baoxin Pill for coronary microvascular function is only in the initial stage and there is still insufficient evidence. In this case, it may waste unnecessary resources to conduct a long-term research to observe clinical end-point. Although the outcomes selected in our study could not provide direct evidence for prognosis, they could still provide some valuable information and lay a foundation for further clinical studies in the future.

CFR, the most commonly used indicator to evaluate coronary microvascular function, refers to the ratio of maximum diastolic period coronary blood flow or myocardial blood flow to the resting period corresponding indexes (Vancheri et al., 2020). At present, CFR less than 2.0 is generally regarded as the main diagnostic criteria for CMD in clinical practice (Ong et al., 2020). At the same time, CFR is often used to evaluate the treatment effect and long-term prognosis of patients with CMD. Studies have shown that a lower CFR was associated with an increased incidence of long-term cardiovascular adverse events and cardiovascular mortality in patients regardless of the presence or absence of obstructive coronary artery disease (Ziadi et al., 2011; Murthy et al., 2014). Therefore, CFR was selected as one of the main outcomes for our review, and the results showed that combination of Shexiang Baoxin Pill and conventional treatment can further increase CFR. Meanwhile, similar results were obtained in subgroups of different treatment duration, age, gender distribution and sample size. However, when the subgroup analysis was conducted based on comorbidity, the result was negative in the subgroup of not comorbid OCAD [MD = 0.54, 95%CI (−0.17, 1.26), *p* = 0.14]. This suggests that the drug may be less effective in improving CFR in patients without OCAD. It should be noted that this subgroup consisted of only two studies, so this conclusion is relatively unreliable and needs to be confirmed by more studies in the future. In addition, we found considerable heterogeneity in the result of this analysis. Although the heterogeneity of some subgroups could be reduced by subgroup analysis, there were still some subgroups with high heterogeneity. We believed that this may be because CFR is susceptible to many factors such as coronary hemodynamics, leading to certain errors in measurement in different research institutions. Therefore, we should take a cautious attitude towards the results of this outcome.

The IMR refers to the ratio of pressure to flow at the distal end of coronary arteries in the state of congestion, which is one of the newer indicators to evaluate coronary microvascular function (Vancheri et al., 2020). Unlike CFR, IMR evaluates coronary microvascular function independently of the epicardial coronary artery, which is less likely to be influenced by coronary hemodynamics and has higher reproducibility (Ng et al., 2006). Therefore, the combined use of IMR and CFR to evaluate coronary microvascular function may have higher accuracy. In 2020, a statement released by the ESC Working Group on Coronary Pathophysiology and Microcirculation put forward that CFR less than 2.0 or IMR greater than 25 both are important markers of coronary microvascular dysfunction (Padro et al., 2020). Similar to CFR, IMR is also associated with patient prognosis. The study found that the value of IMR before percutaneous coronary intervention (PCI) has to do with the incidence of perioperative myocardial infarction (Layland et al., 2012). In patients with acute myocardial infarction, higher IMR indicated an increased incidence of heart failure and all-cause mortality (Carrick et al., 2016). Therefore, IMR was also selected as one of the main outcomes for our review, and the results showed that Shexiang Baoxin Pill could decrease IMR, and consistent conclusions were obtained in subgroups of different treatment duration, age, gender distribution and sample size. As with CFR, negative result was found in the subgroup of not comorbid OCAD [MD = −7.42, 95%CI (−14.72, −0.13), *p* = 0.05]. This further suggests that the drug may be less effective in improving coronary microvascular function in patients without OCAD. But this subgroup also included only two studies, the validity of this conclusion needs to be confirmed by further study. In addition, we found significant heterogeneity in the analysis results of IMR and subgroup analysis just reduced part of the heterogeneity. Since the IMR is a newly discovered indicator in recent years, there may be some differences in the level of measurement technology in different hospitals, we considerd that heterogeneity might result from this.

Dysfunction of vascular endothelial cells resulting in reduced NO production is one of the key pathogenesis of CMD (Gutiérrez et al., 2013). The decrease of NO will cause collagen deposition, leading to microvascular hypertrophy, fibrosis and abnormal diastolic function (Jones et al., 1995; Goligorsky, 2010). Studies have shown that NO-mediated microvascular dilation is significantly impaired in patients with CMD (Kajitani et al., 2019), and a positive correlation has been found between NO bioavailability and CFR (Chen et al., 2002). Thus, it can be seen, serum NO level can reflect coronary microvascular function. The results of our review found that combination of conventional treatment and Shexiang Baoxin Pill can further increase NO compared with conventional treatment alone. This suggests that the drug may improve coronary microvascular function by improving vascular endothelial function. However, on the one hand, when the subgroup analysis was conducted based on average age, we found that Shexiang Baoxin Pill could not increase NO in the subgroups with average age greater than 55 years old [MD = 8.96, 95%CI (−1.85, 19.77), *p* = 0.10]. This suggests that the drug may be less effective in improving vascular endothelial function in older patients. But due to the small sample size, the conclusion cannot be drawn completely at present and needs to be verified by further studies in the future. On the other hand, when the subgroup analysis was conducted based on comorbidity, negative results were obtained in both subgroups [MD = 11.62, 95%CI (−3.54, 26.78), *p* = 0.13] [MD = 12.28, 95%CI (−4.63, 29.20), *p* = 0.15]. Nevertheless, we can still discover a increasing trend of NO in the two subgroups. We thought this result may be related to the small sample size. Once the sample size is enlarged, the conclusion might be reversed. In addition, we found that heterogeneity was significantly reduced when subgroup analysis was performed based on sample size (*I* ^*2*^ = 0%). This shows that sample size may be one of the main source of heterogeneity.

Inflammation can damage to vascular endothelial cells and inhibit endothelium-dependent microvascular dilation, leading to the occurrence of CMD (Zanatta et al., 2019). The hs-CRP is a sensitive and non-specific inflammatory marker, which is one of the commonly used indicators to evaluate the degree of inflammatory response in the body, and its value in the prediction of cardiovascular disease related risk is particularly important. Studies have found that serum hs-CRP levels in patients with CMD are significantly higher than those in healthy people (Aslan et al., 2019), and there is a significant correlation between the serum hs-CRP level and CFR and IMR (Long et al., 2017; Caliskan et al., 2020). Therefore, serum hs-CRP can reflect coronary microvascular function indirectly. Our meta-analysis results shows that combination of Shexiang Baoxin Pill can further decrease hs-CRP and consistent conclusions were obtained in all subgroups, which manifests that the drug may improve coronary microvascular function by inhibiting inflammatory response. Due to the high heterogeneity, subgroup analysis was conducted based on treatment duration, average age, comorbidity and sample size. The result turns out that average age, comorbidity and sample size may be the sources of heterogeneity. But there is still some heterogeneity that can’t be traced, we thought this may be related to differences in detection equipment and techniques between studies.

Although objective indicators are the main criteria for evaluating coronary microvascular function, the remission of clinical symptoms should not be ignored, since one of the purposes of improving coronary microvascular function is to improve patients’ exercise tolerance and living quality. TET is one of the classic methods to evaluate the exercise tolerance of patients with coronary artery disease. It has the advantages of noninvasiveness and simplicity, and has great application value for patients with obstructive coronary artery disease or CMD (Acampa et al., 2016). The results of our review found that combination of conventional treatment and Shexiang Baoxin Pill can’t further increase TET compared with conventional treatment alone [MD = 3.64, 95%CI (−1.17, 8.45), *p =* 0.14]. This seems to demonstrate that the drug failed to improve the exercise endurance of patients with CMD. However, we noted that only two included studies with 116 patients reported TET, which was significantly fewer than other outcomes. Nevertheless, we still found an increasing trend in this outcome. Hence, we consider that this negative result may be related to the small sample size. Once the sample size is enlarged, the conclusion might be reversed. In addition, there is large heterogeneity of this analysis result and it was not possible to conduct subgroup analysis to find sources of heterogeneity due to the small number of included studies. Therefore, we should treat this conclusion with caution.

The pathogenesis of CMD still remains unclear. Currently, endothelial cell dysfunction, resulting in decreased NO release, is recognized as the core pathological process (Gutiérrez et al., 2013). The decrease of NO will not only impair diastolic function of coronary microvasculature, but also lead to microvascular remodeling, which is manifested by rarefaction of microvascular and luminal narrowing (Mohammed et al., 2015; Lindemann et al., 2018). Moreover, inflammatory response and the autonomic nervous system may be involved in the regulation of endothelial cell function, leading to decreased NO release or bioavailability, which could result in the occurrence of CMD as well (Duncker et al., 2015; Zanatta et al., 2019). Because the improvement effect of β-blockers, ACEI, CCB, nitrate, ranolazine and other conventional drugs on coronary microvascular function is not satisfactory (Cannon et al., 1985; Bugiardini et al., 1989; Buus et al., 2004; Mehta et al., 2011; Pauly et al., 2011; Bairey Merz et al., 2016; Zhang et al., 2017), targeted therapy for the pathological process of CMD has been developed in recent years. Some small sample clinical trials of targeting endothelial function or inhibiting inflammatory response had gained some encouraging results, but higher quality studies are needed to confirm these findings (Ikonomidis et al., 2008; Johnson and Gould, 2013). Under the condition that conventional drugs has not been able to obtain satisfactory curative effect, TCM may play a role in the treatment of CMD. Modern pharmacological studies have found that Shexiang Baoxin Pill can improve the function of microvascular endothelial cells and promote microvascular angiogenesis by increasing the expression of endothelial NO synthase and inhibiting the inflammatory response of endothelial cells (Xu et al., 2011; Li et al., 2015). This point suggests that the drug has great potential in improving coronary microvascular function, but its pharmacological mechanism is still not completely clear, which needs more in-depth study in the future.

This study is the first to systematically review the effects of Shexiang Baoxin Pill for coronary microvascular function. There have been three systematic reviews of TCM treatment for coronary microvascular function in the past, but they all had some shortcomings (Wang et al., 2013; Mao et al., 2018; Zhong et al., 2020). First of all, the outcomes selected in these reviews are mainly clinical symptoms, electrocardiogram, blood indicator NO, endothelin-1, hs-CRP etc., while the outcomes that directly reflect coronary microvascular function are lacking, such as CFR, IMR, and so on. In the next place, the treatment duration of most of the studies included in these reviews was about 2 to 4 weeks, which is too short to achieve satisfactory effects. Furthermore, two of these reviews did not define the types of Chinese medicine in the intervention group, leading to a large difference in the intervention measures among the included studies, as well as an unreliable conclusion of the meta-analysis. Compared with the aforementioned reviews, our review included more objective outcomes of coronary microvascular function, the treatment duration of the included studies was longer and the intervention measures were more consistent. Accordingly, the reliability of our conclusions is improved. However, there are still following deficiencies in our study: Firstly, the methodological quality of included studies in our review is not high, the random sequence generation, allocation concealment and blinding method had not been reported in detail, which reduced the reliability of our analysis conclusions to some extent. Secondly, there was a large statistical heterogeneity in the analysis of all outcomes. After comparing the detailed characteristics of each study and conducting subgroup analysis, we discovered that treatment duration, average age, comorbidity, gender distribution and sample size may be the sources of heterogeneity. In addition, no other significant differences was found in clinical and methodological features. We considered the clinical heterogeneity and methodological heterogeneity among these studies to be acceptable, so we applied random effect model to pool the data. However, the large statistical heterogeneity still needs to be paid attention to, suggesting that we should treat the results of this study with more caution. Thirdly, placebos, which often used as control treatment in clinical trials, could influence body’s function by regulating the autonomic nervous system. As we mentioned above, the pathogenesis of CMD may be related to autonomic nervous system dysfunction, and thus the placebo effect should be fully considered when studying the effect of a drug for coronary microvascular function. However, no placebo control was taken of all the included studies in our review. Hence, the effect of Shexiang Baoxin Pill on coronary microvascular function is not completely convincing. Fourthly, the dose of Shexiang Baoxin Pill in all included studies was completely coincident, resulting in an inability to assess the dose-effect relationship of Shexiang Baoxin Pill for coronary microvascular function. Fifthly, the publication bias could not be assessed for the reason that each of the outcomes included less than 10 studies. Sixthly, due to the lack of prognosis outcomes such as cardiovascular adverse events and mortality in the included studies, it was difficult to evaluate the effect of Shexiang Baoxin Pill on the prognosis of CMD patients.

Based on the above discussion, we recommend that future randomized controlled trials of TCM should strictly follow the CONSORT (Consolidated Standards of Reporting Trials) and clinical end-point should be included as far as possible. In addition, we look forward to more high-quality randomized, double-blind controlled trials to provide more sufficient evidence for the Shexiang Baoxin Pill in improving coronary microvascular function.

## Conclusion

Current evidence suggests that Shexiang Baoxin Pill may be effective in the improvement of coronary microvascular function when used in combination with conventional treatment. However, due to the low quality of the included studies, lack of placebo control and high heterogeneity among different studies, we should take a cautious attitude towards this conclusion. Moreover, the safety of Shexiang Baoxin Pill remains uncertain, more high-quality clinical studies are needed to verify the efficacy and safety of this drug in the future.

## Data Availability

The original contributions presented in the study are included in the article/Supplementary Material, further inquiries can be directed to the corresponding authors.
