# Supplementary material for: Effects of Shexiang Baoxin Pill for Coronary Microvascular Function: A Systematic Review and Meta-Analysis
Source: Front Pharmacol. 2021 Nov 2;12:751050. doi: 10.3389/fphar.2021.751050 (PMC8592925; doi:10.3389/fphar.2021.751050)
Supplement: Supplementary file 1 [file Table1.DOCX]

| **SUMMARY TABLE OF THE STUDIES INCLUDED**. | | | | | |
| --- | --- | --- | --- | --- | --- |
| **Study** | **Formulation** | **Source** | **Species, concentration** | **Quality control reported**  **(Y/N)** | **Chemical analysis reported**  **(Y/N)** |
| Chen XL  2016 | Shexiang Baoxin Pill | Shanghai Hutchison Pharmaceuticals Company | - Preputial secretion of *Moschus berezovskii, M. sifanicus or M. moschiferus* [Cervidae; Moschus] concentration uncertainty - Root of *Panax ginseng C.A.Mey.*[Araliaceae; Ginseng Radix et rhizoma] concentration uncertainty - Gall-stone of *Bos taurus domesticus Gmelin*. [Bovidae; Bovis Calculus Artifactus] concentration uncertainty - Bark of *Cinnamomum cassia.* [Lauraceae; Cinnamomi Cortex]   concentration uncertainty   - Secretion from tree trunk of *Liquidambar orientalis Mill.* [Hamamelidaceae R. Br.; Styrax]   concentration uncertainty   - Secretions from skin gland of *Bufo bufo gargarizans* [Bufonidae; Bufonis Venenum]   concentration uncertainty   - Distillation and recrystallization of leaf of *Dryobalanops aromatica C.F.Gaertn.* [Lauraceae; Borneolum Syntheticum]   concentration uncertainty | Y – Prepared according to the Pharmacopoeia of China, 2010 edition | N |
| Fu CH  2021 | Shexiang Baoxin Pill | Shanghai Hutchison Pharmaceuticals Company | - Preputial secretion of *Moschus berezovskii, M. sifanicus or M. moschiferus* [Cervidae; Moschus] concentration uncertainty - Root of *Panax ginseng C.A.Mey.*[Araliaceae; Ginseng Radix et rhizoma] concentration uncertainty - Gall-stone of *Bos taurus domesticus Gmelin*. [Bovidae; Bovis Calculus Artifactus] concentration uncertainty - Bark of *Cinnamomum cassia.* [Lauraceae; Cinnamomi Cortex]   concentration uncertainty   - Secretion from tree trunk of *Liquidambar orientalis Mill.* [Hamamelidaceae R. Br.; Styrax]   concentration uncertainty   - Secretions from skin gland of *Bufo bufo gargarizans* [Bufonidae; Bufonis Venenum]   concentration uncertainty   - Distillation and recrystallization of leaf of *Dryobalanops aromatica C.F.Gaertn.* [Lauraceae; Borneolum Syntheticum]   concentration uncertainty | Y – Prepared according to the Pharmacopoeia of China, 2010 edition | N |
| Shen SX  2021 | Shexiang Baoxin Pill | Shanghai Hutchison Pharmaceuticals Company | - Preputial secretion of *Moschus berezovskii, M. sifanicus or M. moschiferus* [Cervidae; Moschus] concentration uncertainty - Root of *Panax ginseng C.A.Mey.*[Araliaceae; Ginseng Radix et rhizoma] concentration uncertainty - Gall-stone of *Bos taurus domesticus Gmelin*. [Bovidae; Bovis Calculus Artifactus] concentration uncertainty - Bark of *Cinnamomum cassia.* [Lauraceae; Cinnamomi Cortex]   concentration uncertainty   - Secretion from tree trunk of *Liquidambar orientalis Mill.* [Hamamelidaceae R. Br.; Styrax]   concentration uncertainty   - Secretions from skin gland of *Bufo bufo gargarizans* [Bufonidae; Bufonis Venenum]   concentration uncertainty   - Distillation and recrystallization of leaf of *Dryobalanops aromatica C.F.Gaertn.* [Lauraceae; Borneolum Syntheticum]   concentration uncertainty | Y – Prepared according to the Pharmacopoeia of China, 2010 edition | N |
| Song KY  2011 | Shexiang Baoxin Pill | Shanghai Hutchison Pharmaceuticals Company | - Preputial secretion of *Moschus berezovskii, M. sifanicus or M. moschiferus* [Cervidae; Moschus] concentration uncertainty - Root of *Panax ginseng C.A.Mey.*[Araliaceae; Ginseng Radix et rhizoma] concentration uncertainty - Gall-stone of *Bos taurus domesticus Gmelin*. [Bovidae; Bovis Calculus Artifactus] concentration uncertainty - Bark of *Cinnamomum cassia.* [Lauraceae; Cinnamomi Cortex]   concentration uncertainty   - Secretion from tree trunk of *Liquidambar orientalis Mill.* [Hamamelidaceae R. Br.; Styrax]   concentration uncertainty   - Secretions from skin gland of *Bufo bufo gargarizans* [Bufonidae; Bufonis Venenum]   concentration uncertainty   - Distillation and recrystallization of leaf of *Dryobalanops aromatica C.F.Gaertn.* [Lauraceae; Borneolum Syntheticum]   concentration uncertainty | Y – Prepared according to the Pharmacopoeia of China, 2010 edition | N |
| Song Z  2021 | Shexiang Baoxin Pill | Shanghai Hutchison Pharmaceuticals Company | - Preputial secretion of *Moschus berezovskii, M. sifanicus or M. moschiferus* [Cervidae; Moschus] concentration uncertainty - Root of *Panax ginseng C.A.Mey.*[Araliaceae; Ginseng Radix et rhizoma] concentration uncertainty - Gall-stone of *Bos taurus domesticus Gmelin*. [Bovidae; Bovis Calculus Artifactus] concentration uncertainty - Bark of *Cinnamomum cassia.* [Lauraceae; Cinnamomi Cortex]   concentration uncertainty   - Secretion from tree trunk of *Liquidambar orientalis Mill.* [Hamamelidaceae R. Br.; Styrax]   concentration uncertainty   - Secretions from skin gland of *Bufo bufo gargarizans* [Bufonidae; Bufonis Venenum]   concentration uncertainty   - Distillation and recrystallization of leaf of *Dryobalanops aromatica C.F.Gaertn.* [Lauraceae; Borneolum Syntheticum]   concentration uncertainty | Y – Prepared according to the Pharmacopoeia of China, 2010 edition | N |
| Sun XY  2020 | Shexiang Baoxin Pill | Shanghai Hutchison Pharmaceuticals Company | - Preputial secretion of *Moschus berezovskii, M. sifanicus or M. moschiferus* [Cervidae; Moschus] concentration uncertainty - Root of *Panax ginseng C.A.Mey.*[Araliaceae; Ginseng Radix et rhizoma] concentration uncertainty - Gall-stone of *Bos taurus domesticus Gmelin*. [Bovidae; Bovis Calculus Artifactus] concentration uncertainty - Bark of *Cinnamomum cassia.* [Lauraceae; Cinnamomi Cortex]   concentration uncertainty   - Secretion from tree trunk of *Liquidambar orientalis Mill.* [Hamamelidaceae R. Br.; Styrax]   concentration uncertainty   - Secretions from skin gland of *Bufo bufo gargarizans* [Bufonidae; Bufonis Venenum]   concentration uncertainty   - Distillation and recrystallization of leaf of *Dryobalanops aromatica C.F.Gaertn.* [Lauraceae; Borneolum Syntheticum]   concentration uncertainty | Y – Prepared according to the Pharmacopoeia of China, 2010 edition | N |
| Wang HZ  2015 | Shexiang Baoxin Pill | Shanghai Hutchison Pharmaceuticals Company | - Preputial secretion of *Moschus berezovskii, M. sifanicus or M. moschiferus* [Cervidae; Moschus] concentration uncertainty - Root of *Panax ginseng C.A.Mey.*[Araliaceae; Ginseng Radix et rhizoma] concentration uncertainty - Gall-stone of *Bos taurus domesticus Gmelin*. [Bovidae; Bovis Calculus Artifactus] concentration uncertainty - Bark of *Cinnamomum cassia.* [Lauraceae; Cinnamomi Cortex]   concentration uncertainty   - Secretion from tree trunk of *Liquidambar orientalis Mill.* [Hamamelidaceae R. Br.; Styrax]   concentration uncertainty   - Secretions from skin gland of *Bufo bufo gargarizans* [Bufonidae; Bufonis Venenum]   concentration uncertainty   - Distillation and recrystallization of leaf of *Dryobalanops aromatica C.F.Gaertn.* [Lauraceae; Borneolum Syntheticum]   concentration uncertainty | Y – Prepared according to the Pharmacopoeia of China, 2010 edition | N |
| Wu CY  2019 | Shexiang Baoxin Pill | Shanghai Hutchison Pharmaceuticals Company | - Preputial secretion of *Moschus berezovskii, M. sifanicus or M. moschiferus* [Cervidae; Moschus] concentration uncertainty - Root of *Panax ginseng C.A.Mey.*[Araliaceae; Ginseng Radix et rhizoma] concentration uncertainty - Gall-stone of *Bos taurus domesticus Gmelin*. [Bovidae; Bovis Calculus Artifactus] concentration uncertainty - Bark of *Cinnamomum cassia.* [Lauraceae; Cinnamomi Cortex]   concentration uncertainty   - Secretion from tree trunk of *Liquidambar orientalis Mill.* [Hamamelidaceae R. Br.; Styrax]   concentration uncertainty   - Secretions from skin gland of *Bufo bufo gargarizans* [Bufonidae; Bufonis Venenum]   concentration uncertainty   - Distillation and recrystallization of leaf of *Dryobalanops aromatica C.F.Gaertn.* [Lauraceae; Borneolum Syntheticum]   concentration uncertainty | Y – Prepared according to the Pharmacopoeia of China, 2010 edition | N |
| Yan XY  2021 | Shexiang Baoxin Pill | Shanghai Hutchison Pharmaceuticals Company | - Preputial secretion of *Moschus berezovskii, M. sifanicus or M. moschiferus* [Cervidae; Moschus] concentration uncertainty - Root of *Panax ginseng C.A.Mey.*[Araliaceae; Ginseng Radix et rhizoma] concentration uncertainty - Gall-stone of *Bos taurus domesticus Gmelin*. [Bovidae; Bovis Calculus Artifactus] concentration uncertainty - Bark of *Cinnamomum cassia.* [Lauraceae; Cinnamomi Cortex]   concentration uncertainty   - Secretion from tree trunk of *Liquidambar orientalis Mill.* [Hamamelidaceae R. Br.; Styrax]   concentration uncertainty   - Secretions from skin gland of *Bufo bufo gargarizans* [Bufonidae; Bufonis Venenum]   concentration uncertainty   - Distillation and recrystallization of leaf of *Dryobalanops aromatica C.F.Gaertn.* [Lauraceae; Borneolum Syntheticum]   concentration uncertainty | Y – Prepared according to the Pharmacopoeia of China, 2010 edition | N |
| Zhang HF  2020 | Shexiang Baoxin Pill | Shanghai Hutchison Pharmaceuticals Company | - Preputial secretion of *Moschus berezovskii, M. sifanicus or M. moschiferus* [Cervidae; Moschus] concentration uncertainty - Root of *Panax ginseng C.A.Mey.*[Araliaceae; Ginseng Radix et rhizoma] concentration uncertainty - Gall-stone of *Bos taurus domesticus Gmelin*. [Bovidae; Bovis Calculus Artifactus] concentration uncertainty - Bark of *Cinnamomum cassia.* [Lauraceae; Cinnamomi Cortex]   concentration uncertainty   - Secretion from tree trunk of *Liquidambar orientalis Mill.* [Hamamelidaceae R. Br.; Styrax]   concentration uncertainty   - Secretions from skin gland of *Bufo bufo gargarizans* [Bufonidae; Bufonis Venenum]   concentration uncertainty   - Distillation and recrystallization of leaf of *Dryobalanops aromatica C.F.Gaertn.* [Lauraceae; Borneolum Syntheticum]   concentration uncertainty | Y – Prepared according to the Pharmacopoeia of China, 2010 edition | N |
| Zhang LW  2019 | Shexiang Baoxin Pill | Shanghai Hutchison Pharmaceuticals Company | - Preputial secretion of *Moschus berezovskii, M. sifanicus or M. moschiferus* [Cervidae; Moschus] concentration uncertainty - Root of *Panax ginseng C.A.Mey.*[Araliaceae; Ginseng Radix et rhizoma] concentration uncertainty - Gall-stone of *Bos taurus domesticus Gmelin*. [Bovidae; Bovis Calculus Artifactus] concentration uncertainty - Bark of *Cinnamomum cassia.* [Lauraceae; Cinnamomi Cortex]   concentration uncertainty   - Secretion from tree trunk of *Liquidambar orientalis Mill.* [Hamamelidaceae R. Br.; Styrax]   concentration uncertainty   - Secretions from skin gland of *Bufo bufo gargarizans* [Bufonidae; Bufonis Venenum]   concentration uncertainty   - Distillation and recrystallization of leaf of *Dryobalanops aromatica C.F.Gaertn.* [Lauraceae; Borneolum Syntheticum]   concentration uncertainty | Y – Prepared according to the Pharmacopoeia of China, 2010 edition | N |
