# Supplementary figures and images for: Effects of Shexiang Baoxin Pill for Coronary Microvascular Function: A Systematic Review and Meta-Analysis
Source: Front Pharmacol. 2021 Nov 2;12:751050. doi: 10.3389/fphar.2021.751050 (PMC8592925; doi:10.3389/fphar.2021.751050)

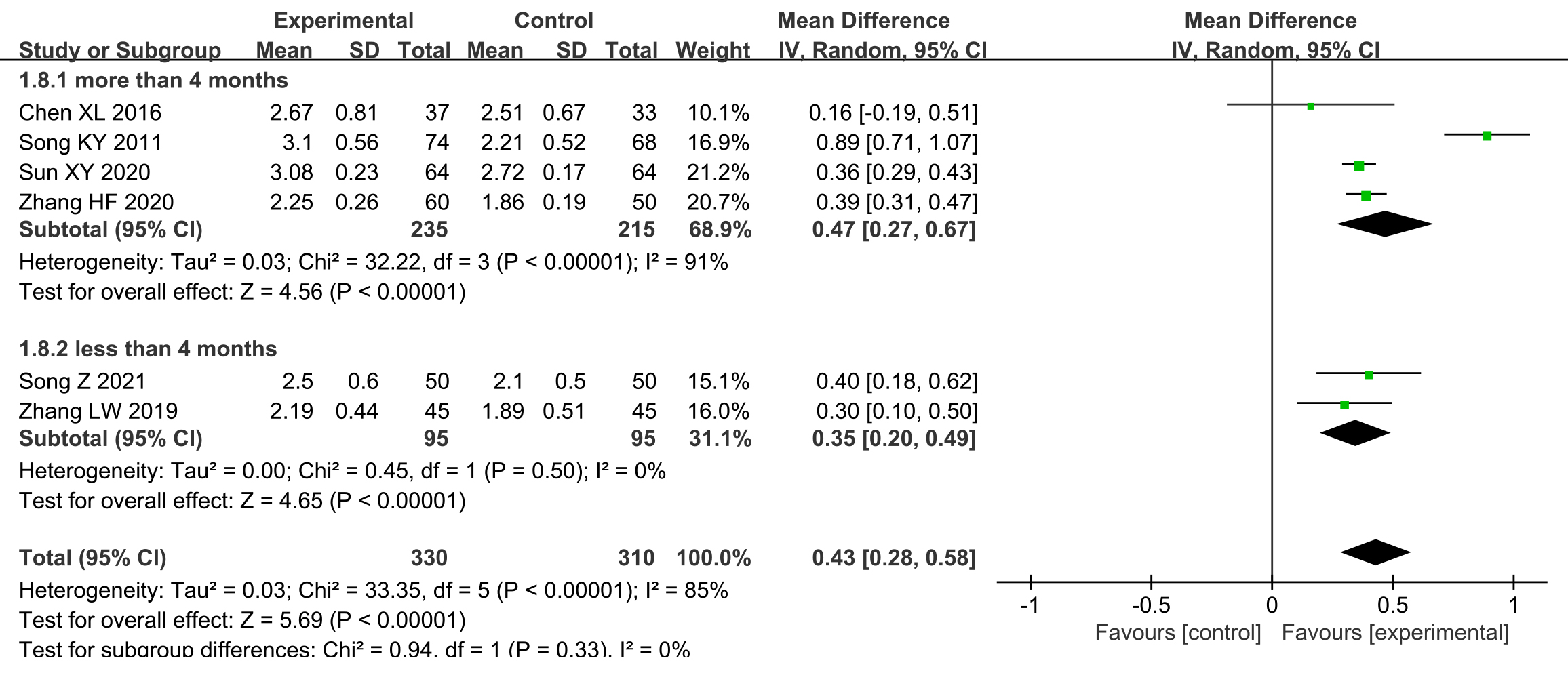

Supplement: Supplementary file 2 [file DataSheet1.ZIP › Supplementary Figure/Supplementary Figure 1.jpg]

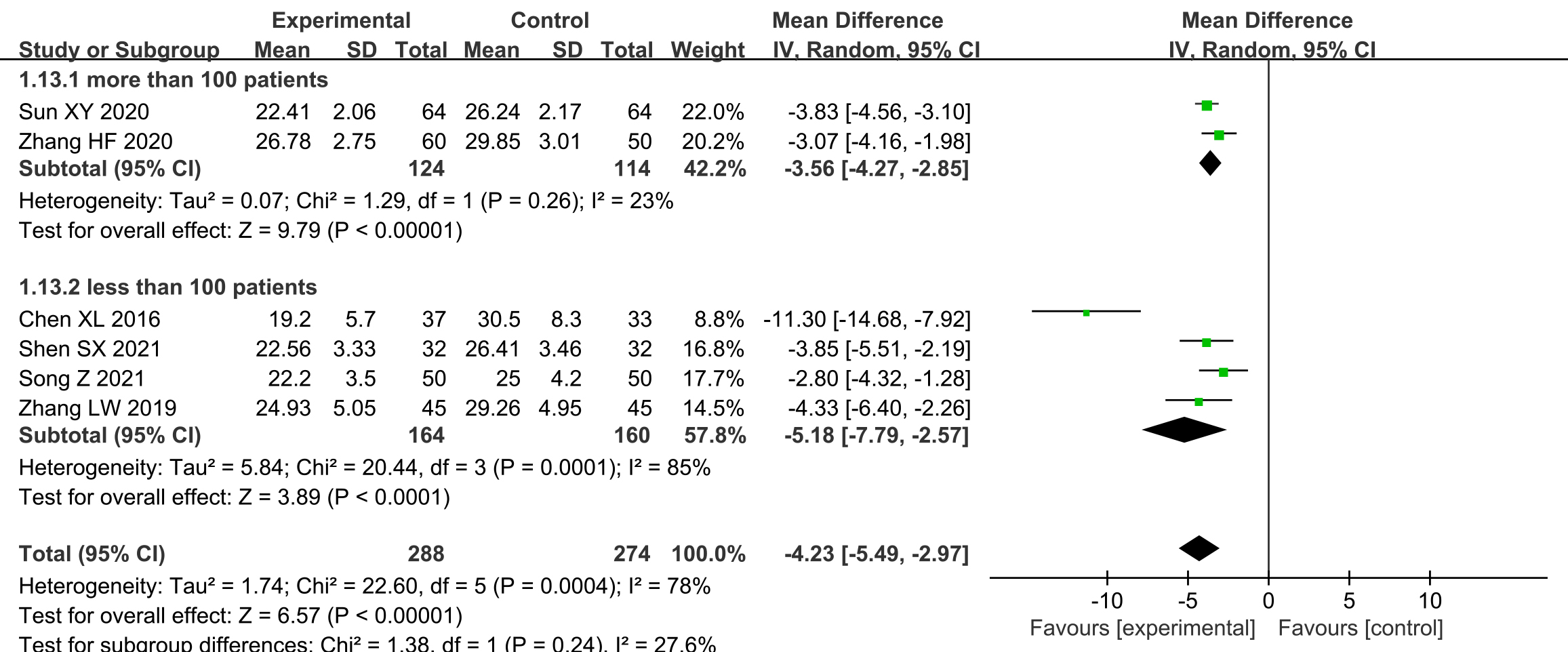

Supplement: Supplementary file 2 [file DataSheet1.ZIP › Supplementary Figure/Supplementary Figure 10.jpg]

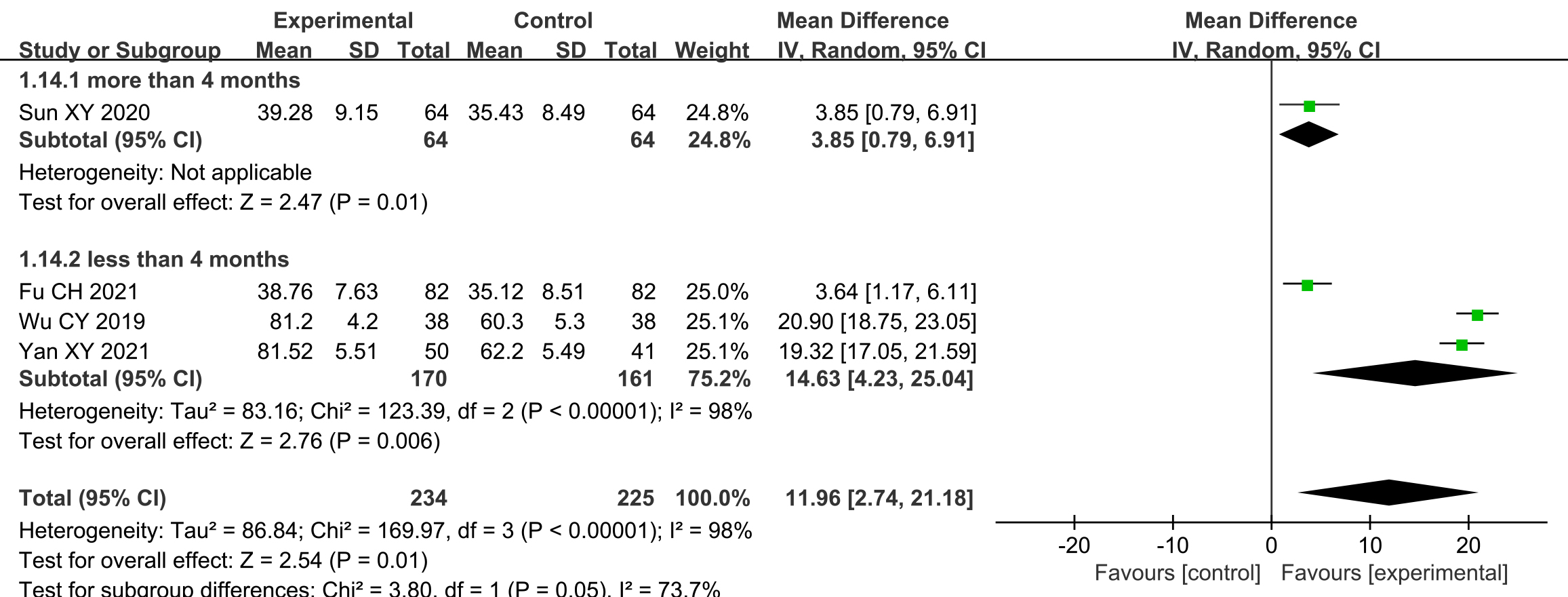

Supplement: Supplementary file 2 [file DataSheet1.ZIP › Supplementary Figure/Supplementary Figure 11.jpg]

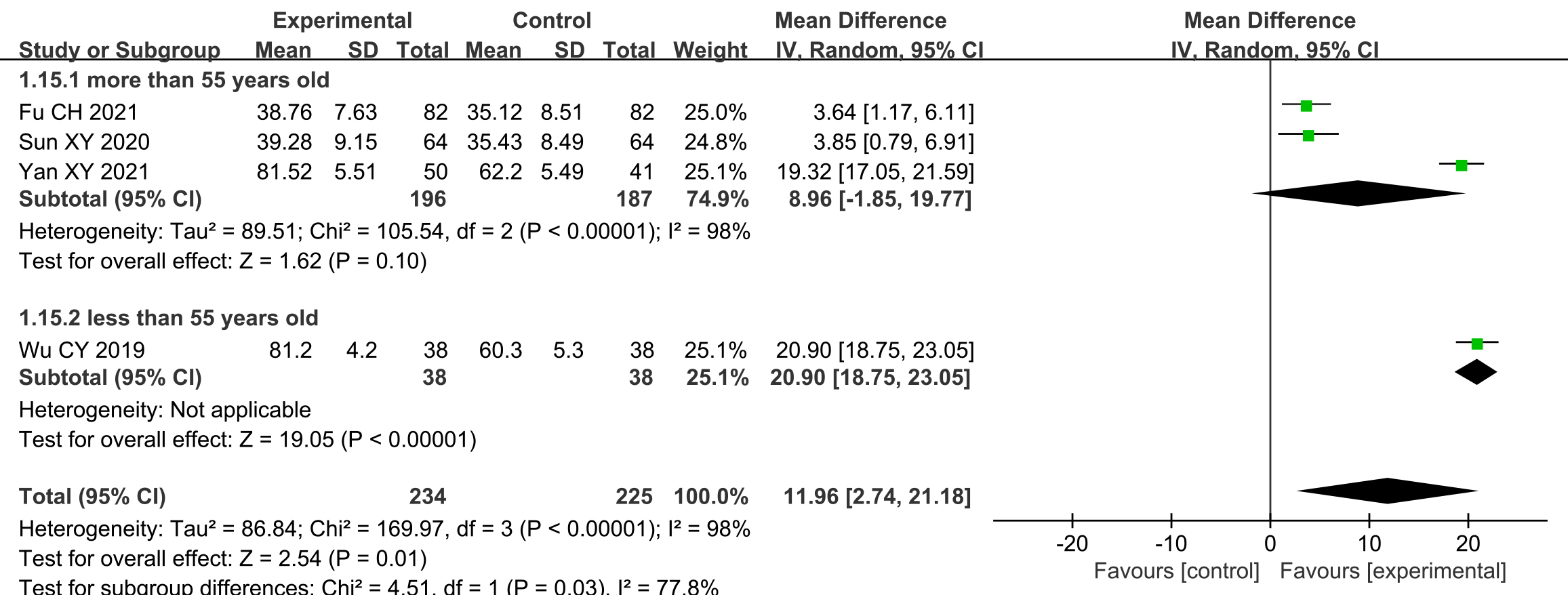

Supplement: Supplementary file 2 [file DataSheet1.ZIP › Supplementary Figure/Supplementary Figure 12.jpg]

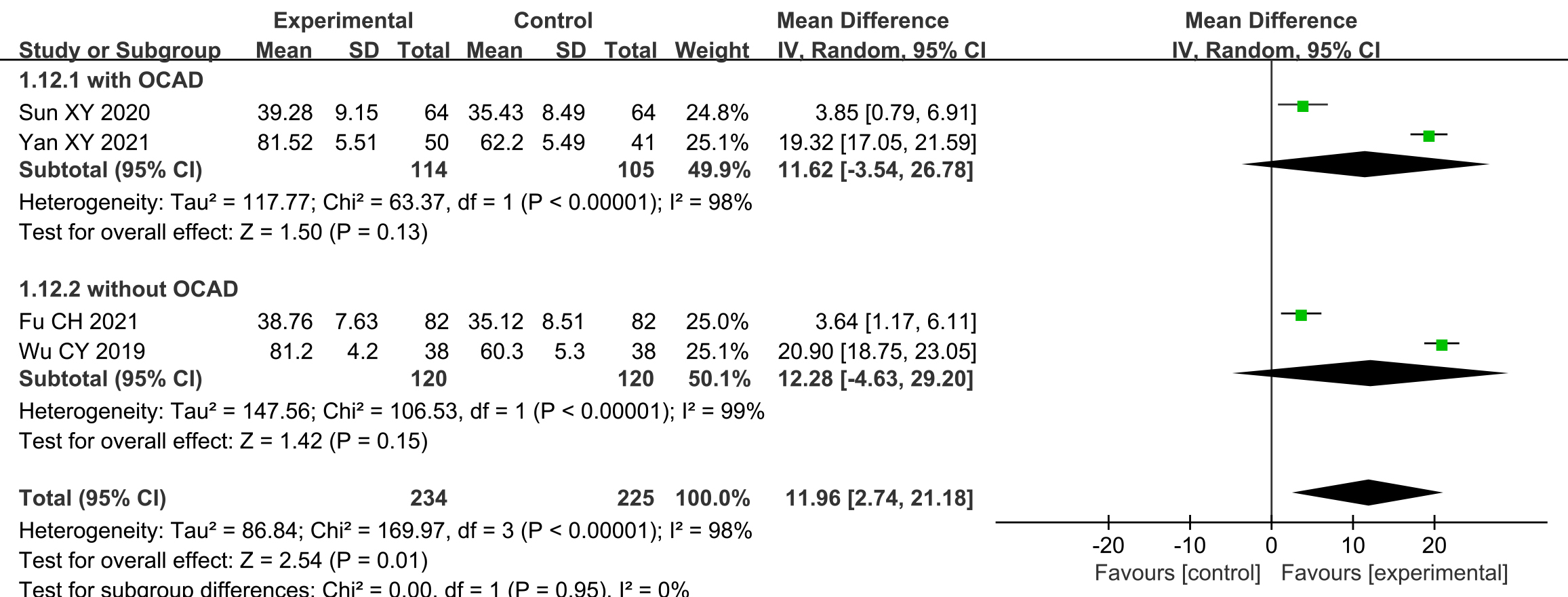

Supplement: Supplementary file 2 [file DataSheet1.ZIP › Supplementary Figure/Supplementary Figure 13.jpg]

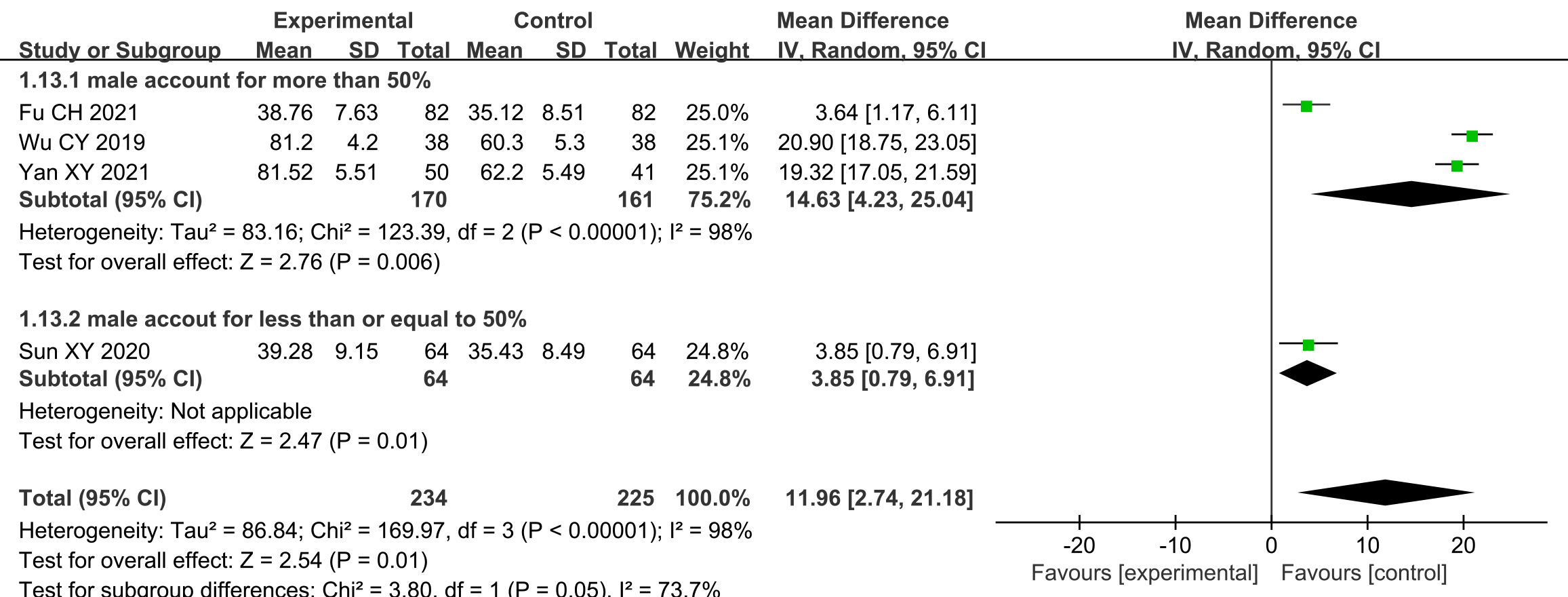

Supplement: Supplementary file 2 [file DataSheet1.ZIP › Supplementary Figure/Supplementary Figure 14.jpg]

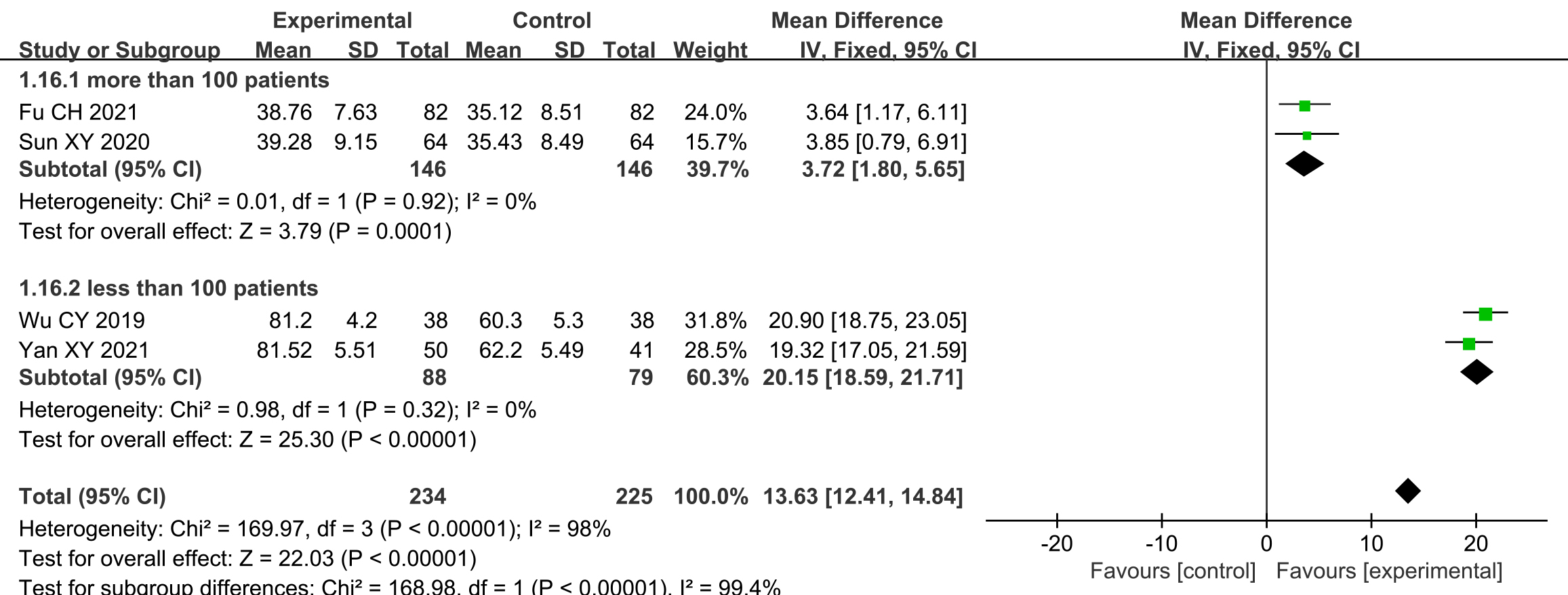

Supplement: Supplementary file 2 [file DataSheet1.ZIP › Supplementary Figure/Supplementary Figure 15.jpg]

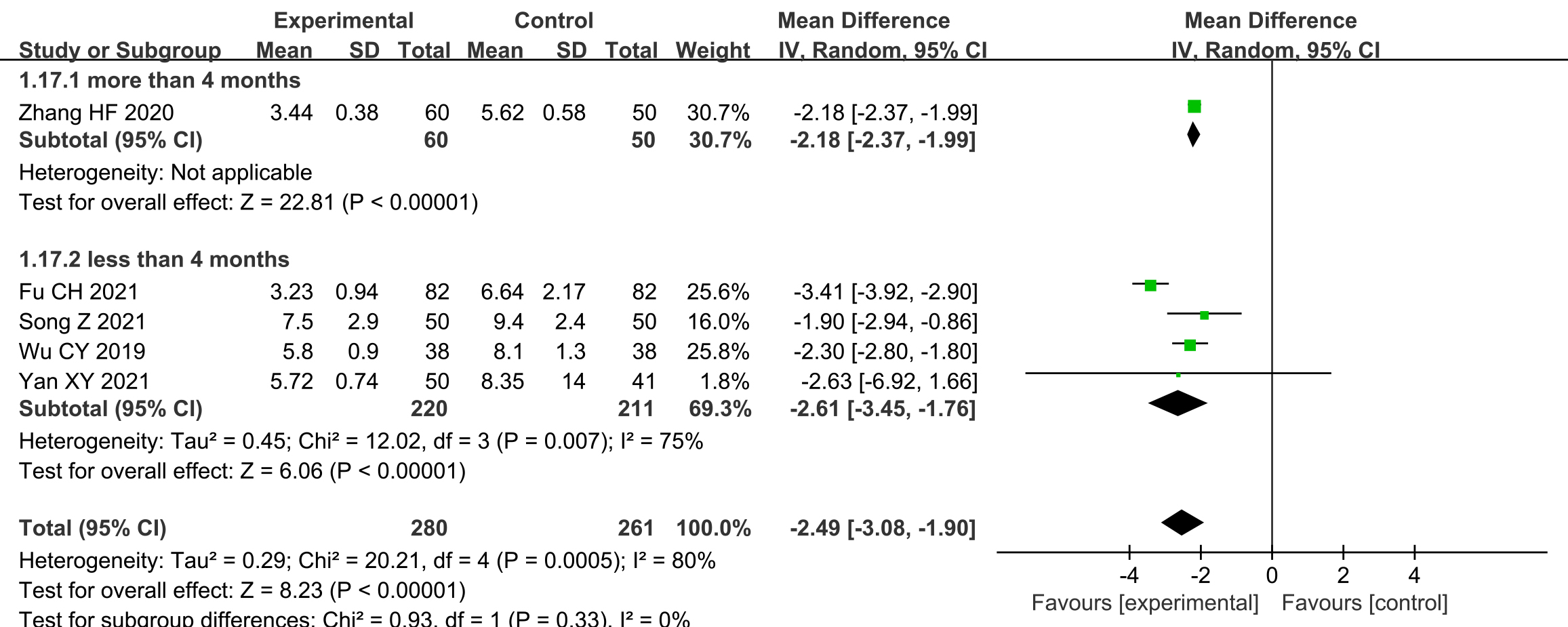

Supplement: Supplementary file 2 [file DataSheet1.ZIP › Supplementary Figure/Supplementary Figure 16.jpg]

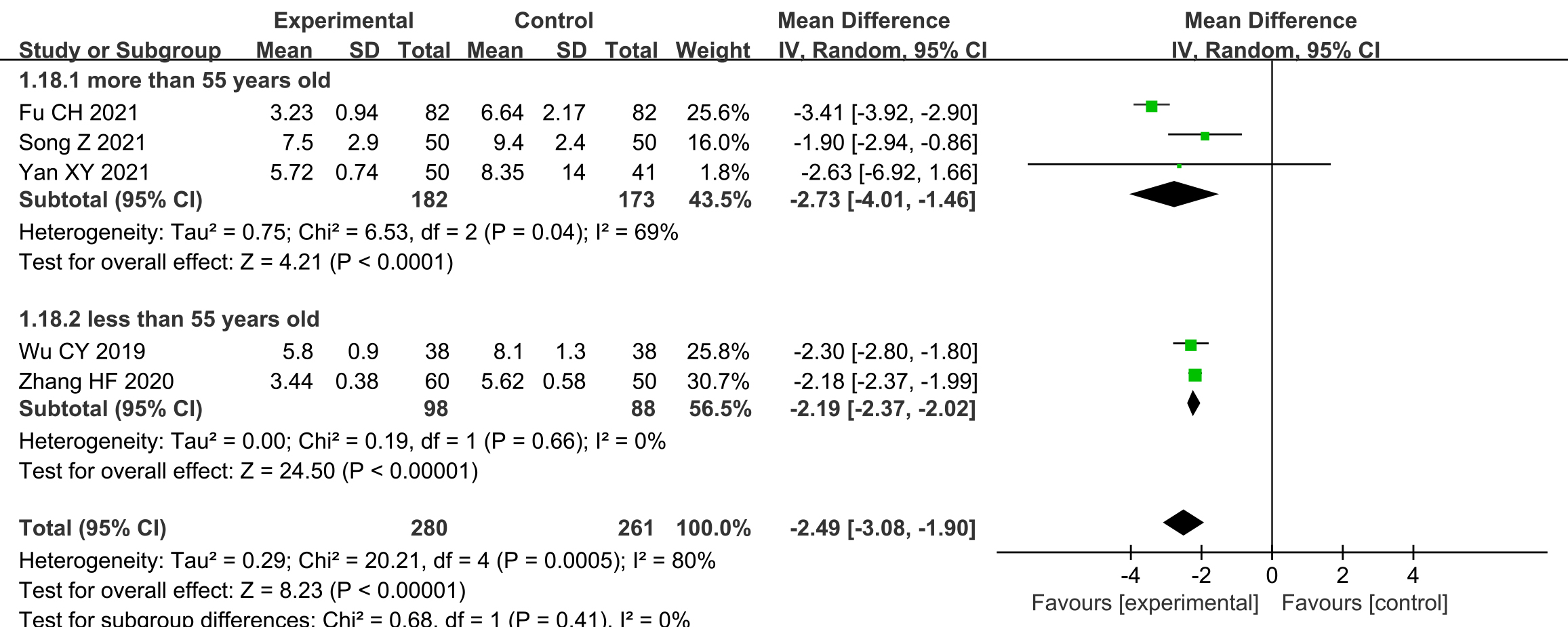

Supplement: Supplementary file 2 [file DataSheet1.ZIP › Supplementary Figure/Supplementary Figure 17.jpg]

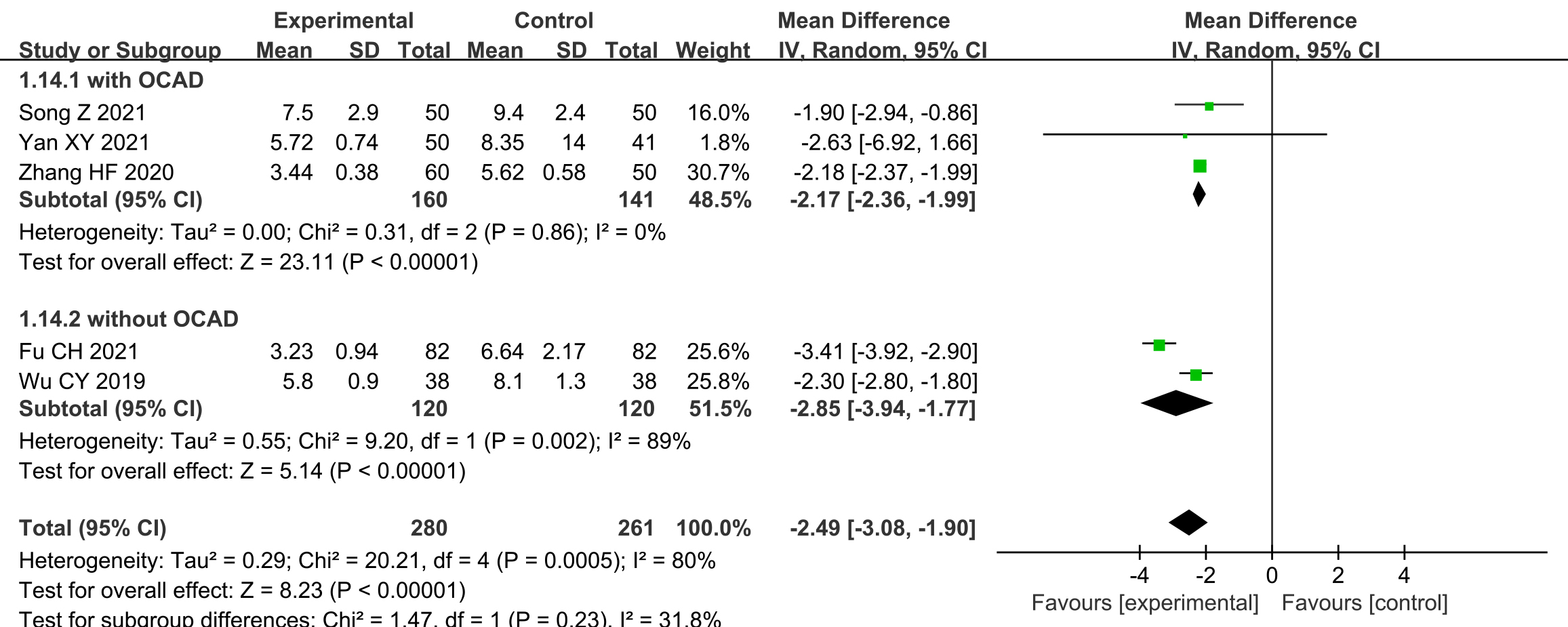

Supplement: Supplementary file 2 [file DataSheet1.ZIP › Supplementary Figure/Supplementary Figure 18.jpg]

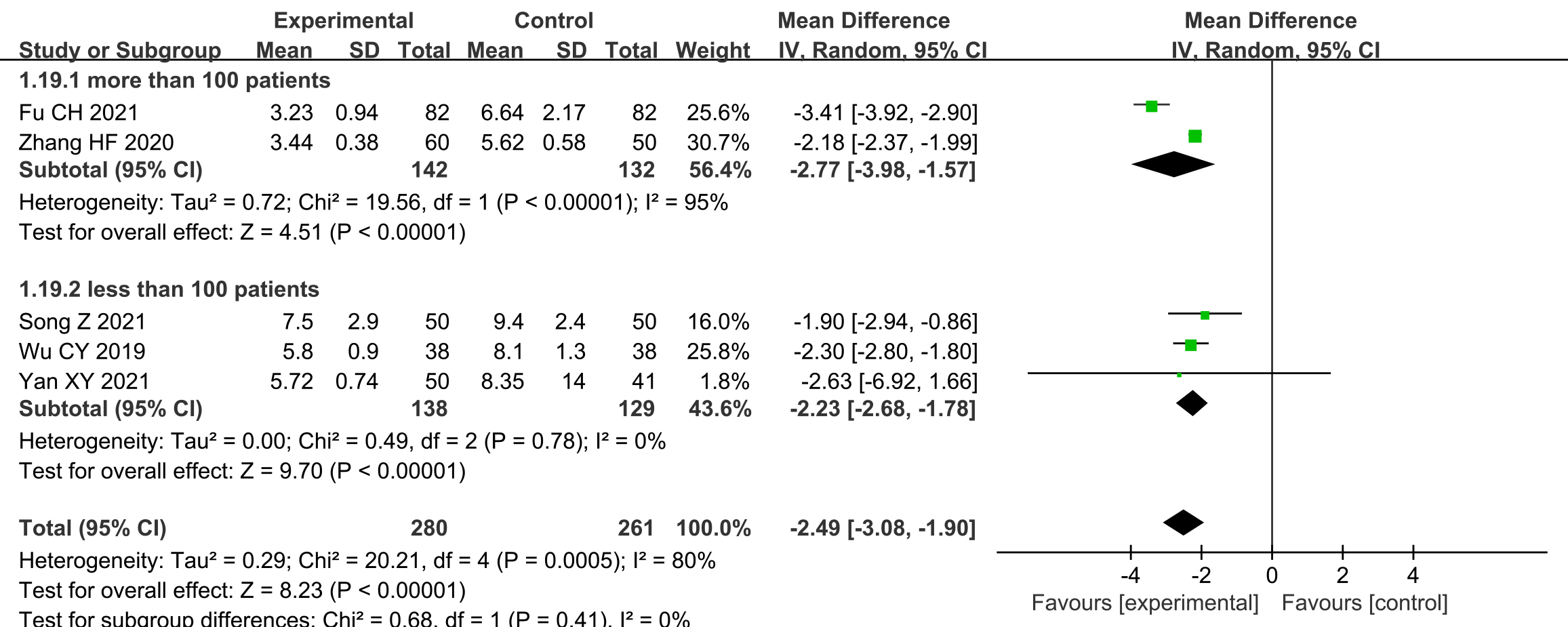

Supplement: Supplementary file 2 [file DataSheet1.ZIP › Supplementary Figure/Supplementary Figure 19.jpg]

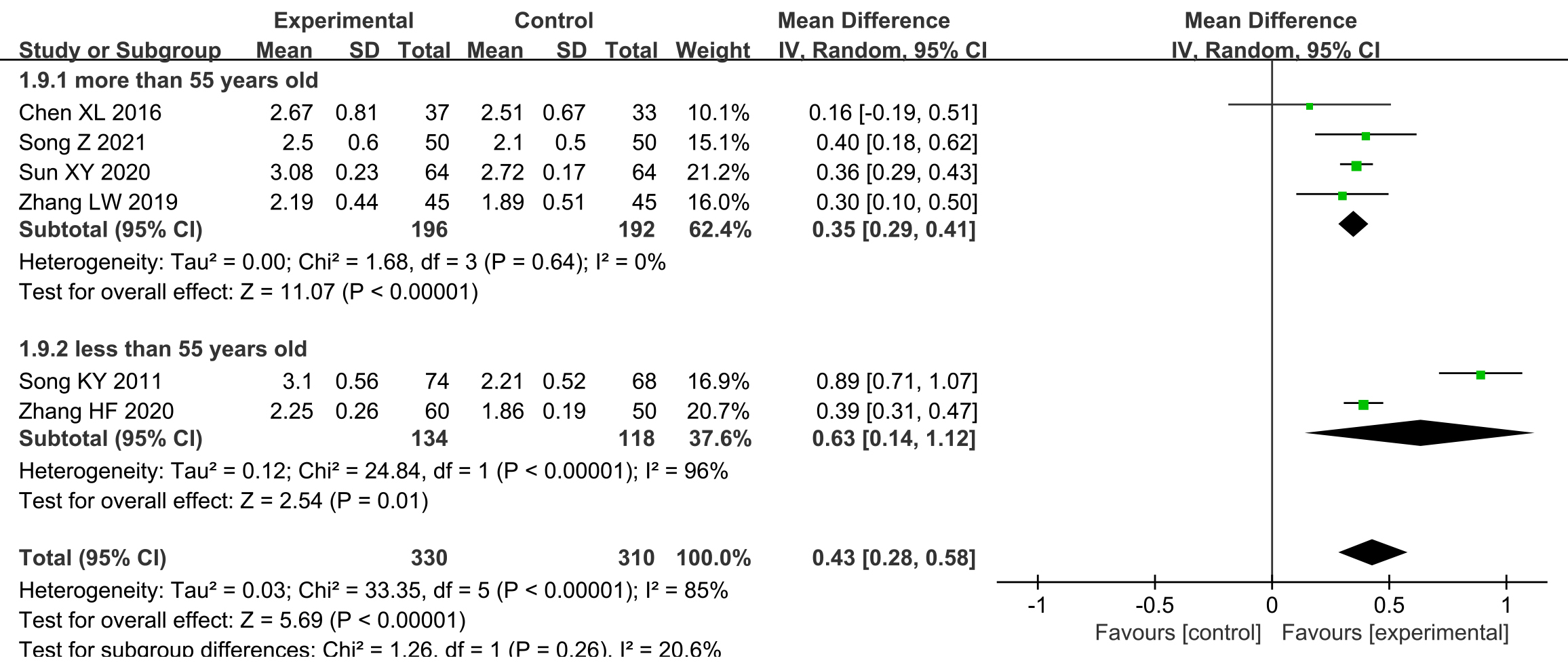

Supplement: Supplementary file 2 [file DataSheet1.ZIP › Supplementary Figure/Supplementary Figure 2.jpg]

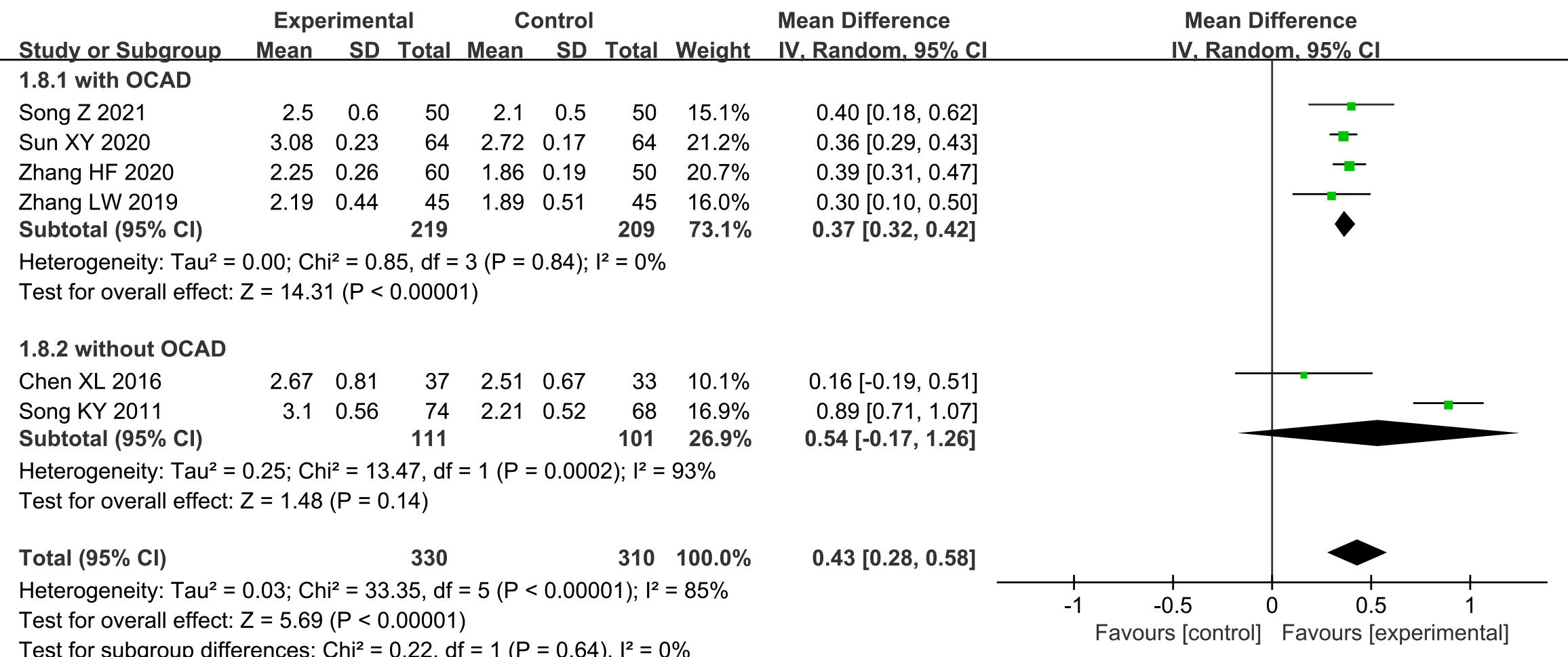

Supplement: Supplementary file 2 [file DataSheet1.ZIP › Supplementary Figure/Supplementary Figure 3.jpg]

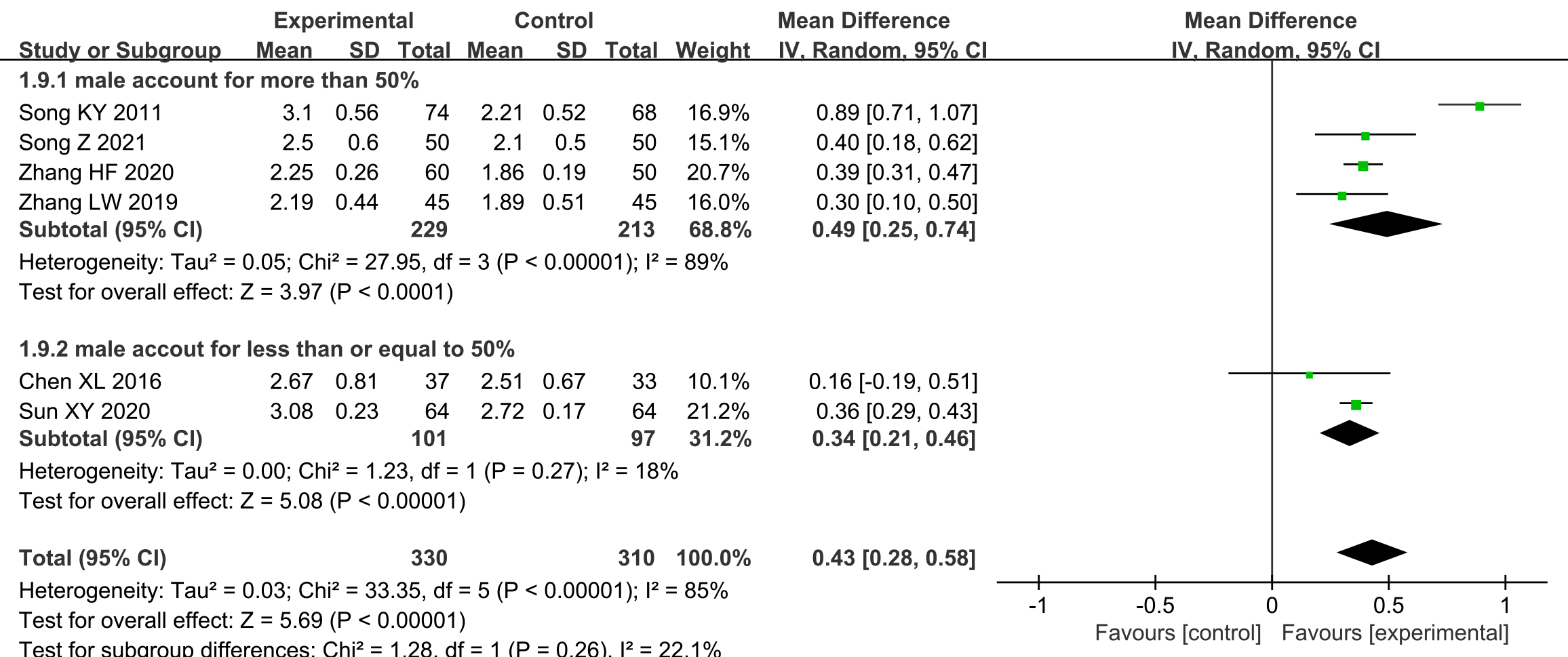

Supplement: Supplementary file 2 [file DataSheet1.ZIP › Supplementary Figure/Supplementary Figure 4.jpg]

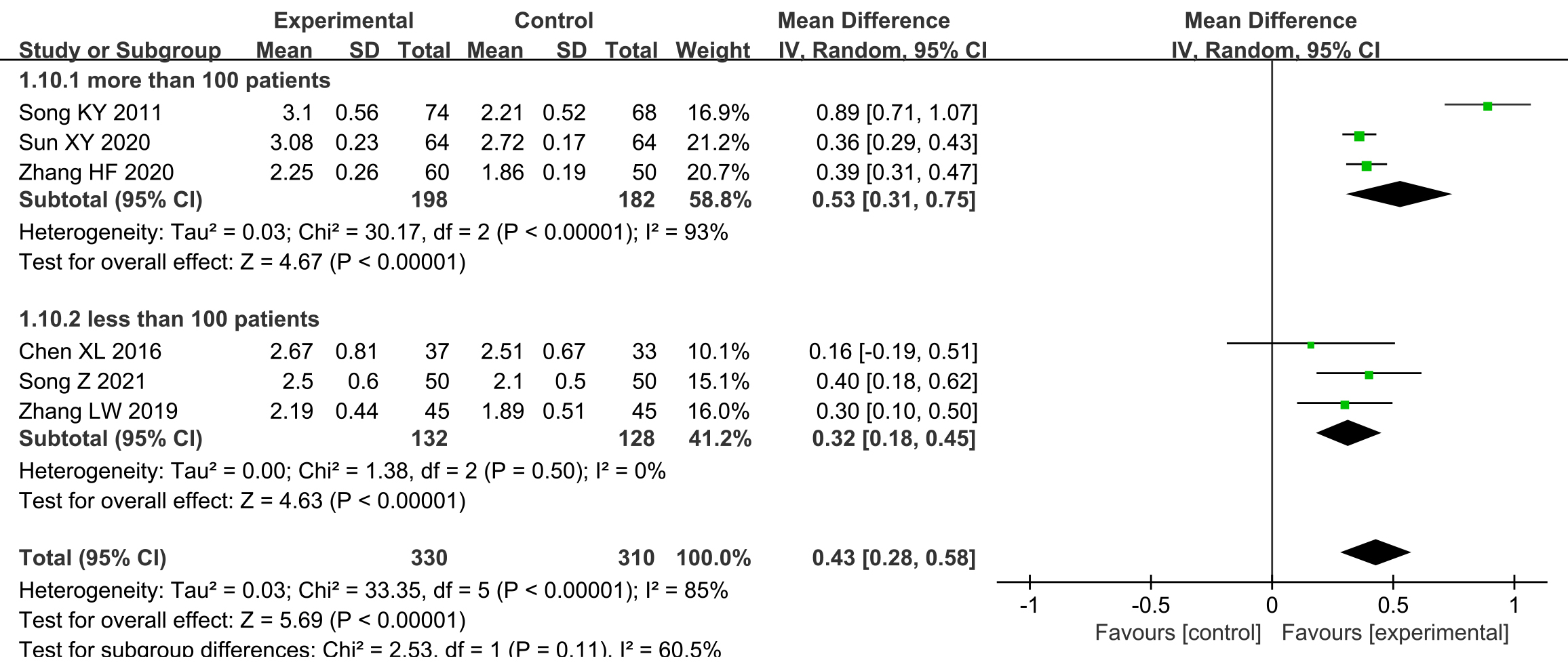

Supplement: Supplementary file 2 [file DataSheet1.ZIP › Supplementary Figure/Supplementary Figure 5.jpg]

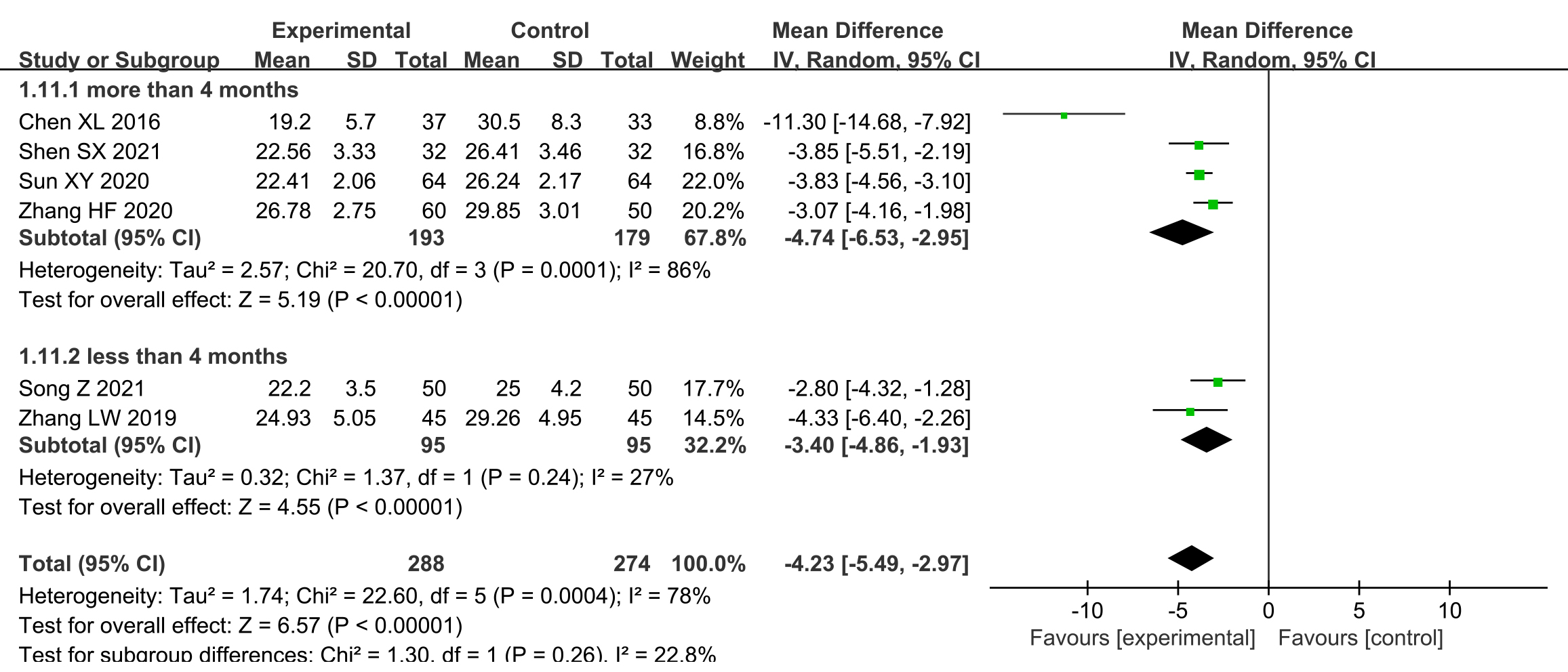

Supplement: Supplementary file 2 [file DataSheet1.ZIP › Supplementary Figure/Supplementary Figure 6.jpg]

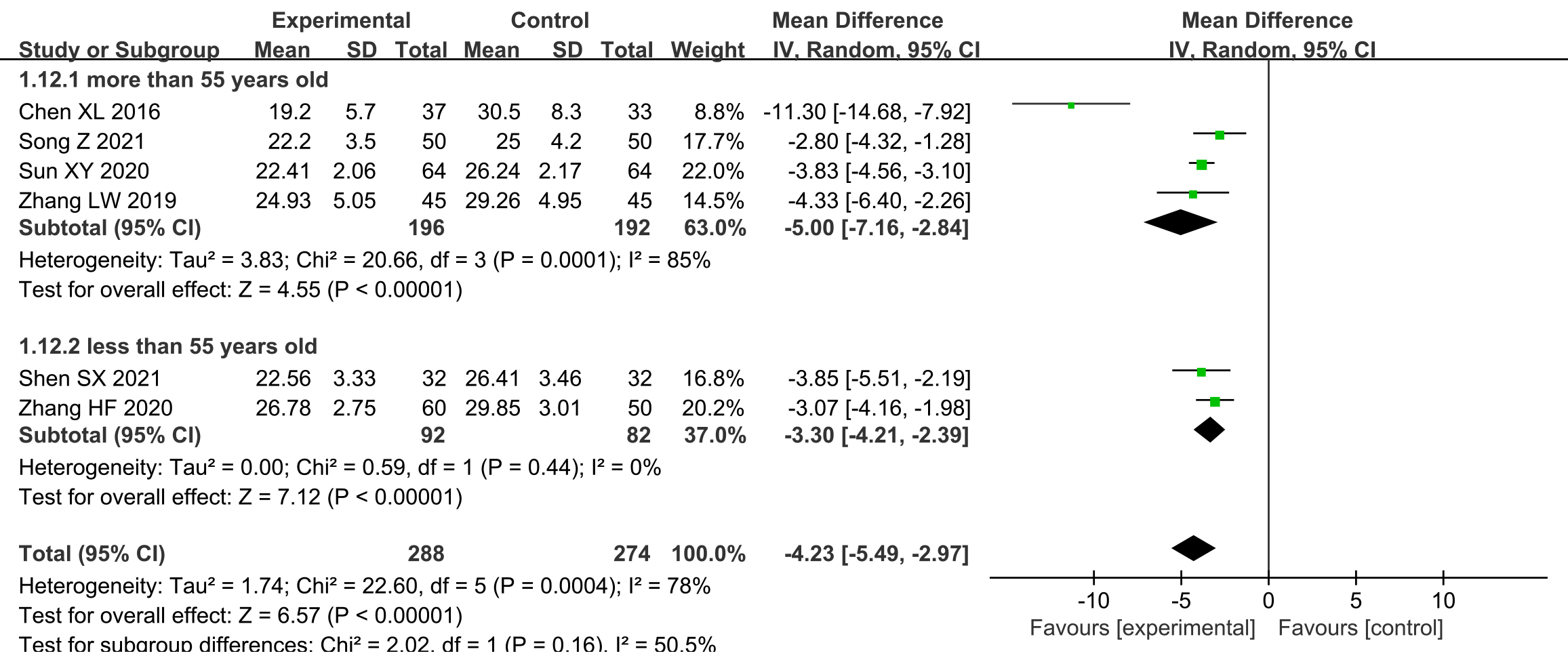

Supplement: Supplementary file 2 [file DataSheet1.ZIP › Supplementary Figure/Supplementary Figure 7.jpg]

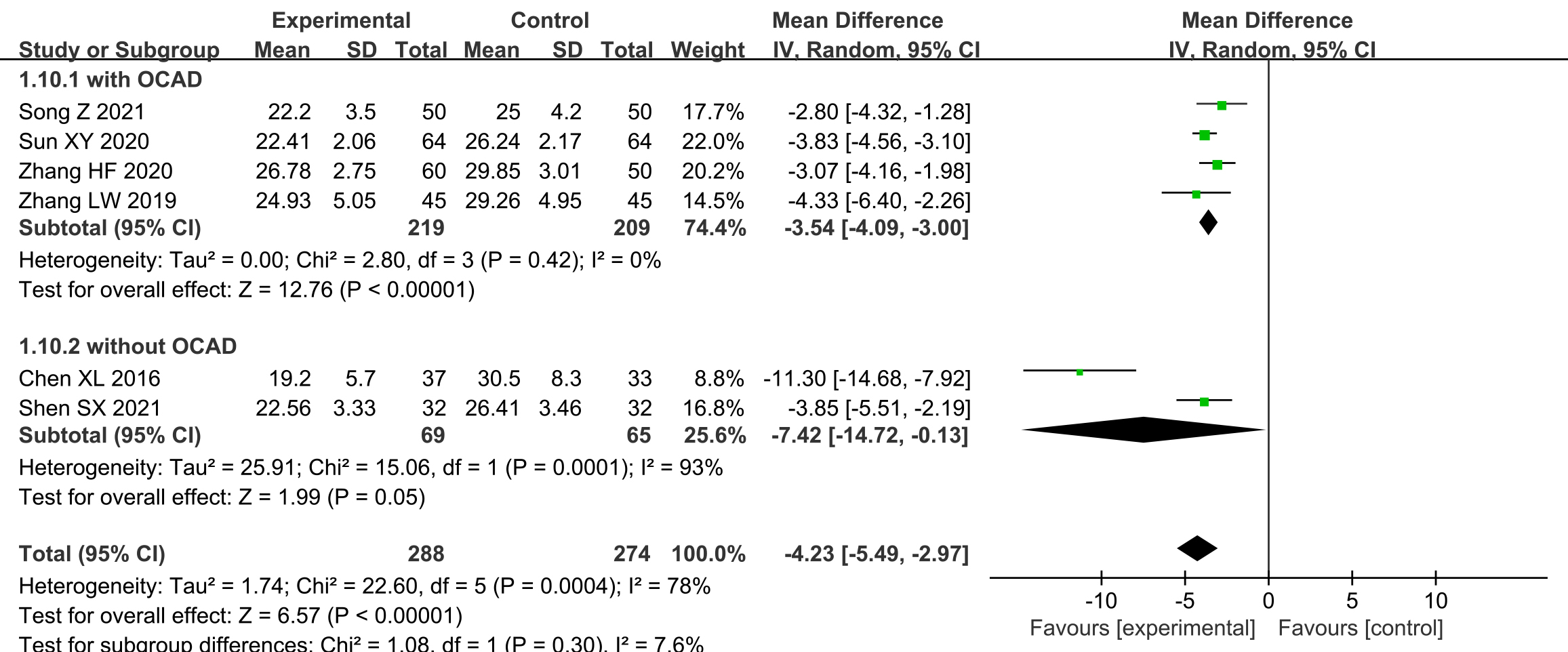

Supplement: Supplementary file 2 [file DataSheet1.ZIP › Supplementary Figure/Supplementary Figure 8.jpg]

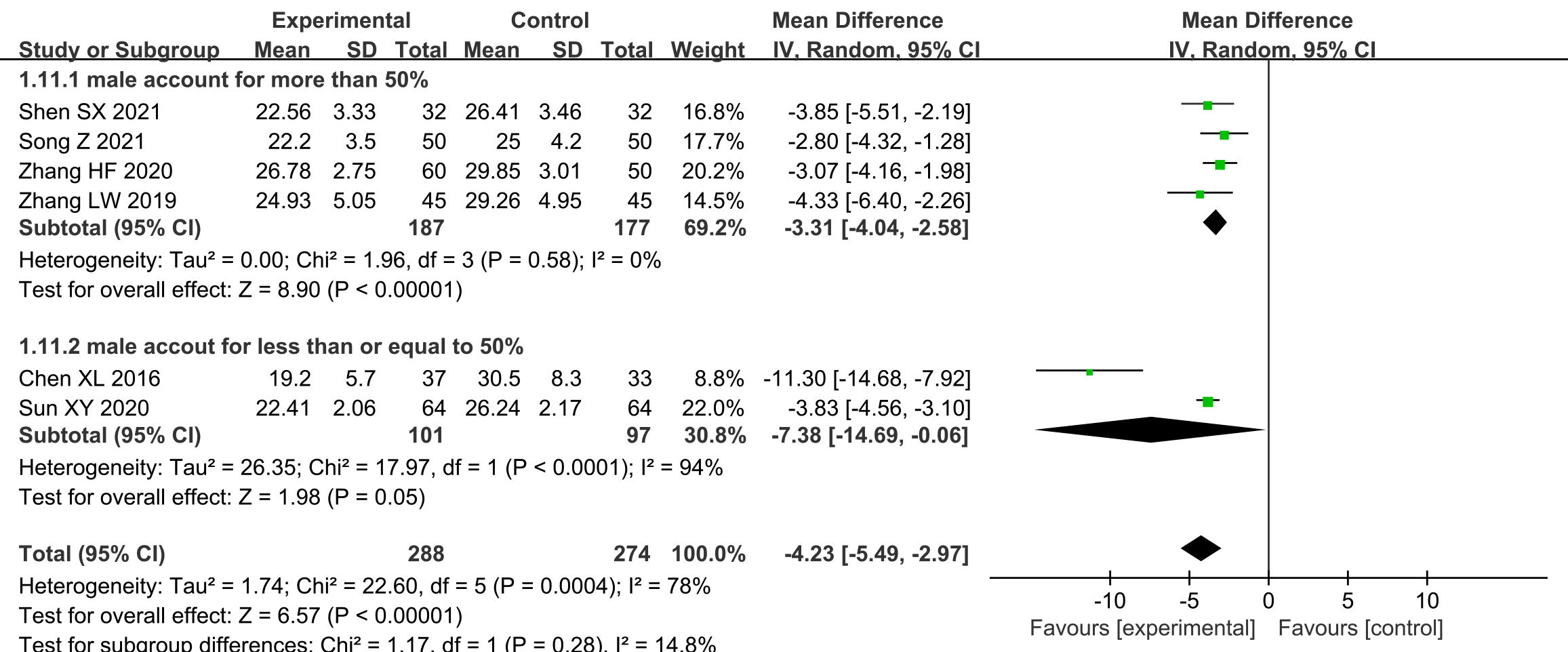

Supplement: Supplementary file 2 [file DataSheet1.ZIP › Supplementary Figure/Supplementary Figure 9.jpg]
