# Supplementary material for: Effects of Shexiang Baoxin Pill for Coronary Microvascular Function: A Systematic Review and Meta-Analysis
Source: Front Pharmacol. 2021 Nov 2;12:751050. doi: 10.3389/fphar.2021.751050 (PMC8592925; doi:10.3389/fphar.2021.751050)
Supplement: Supplementary file 3 [file Table2.DOCX]

Chen XL 2016

| **Domain** | **Signalling question** | **Response** | **Comments** |
| --- | --- | --- | --- |
| **Bias arising from the randomization process** | 1.1 Was the allocation sequence random? | NI | The authors did not report specific randomization methods. |
|  | 1.2 Was the allocation sequence concealed until participants were enrolled and assigned to interventions? | NI | The authors did not report specific allocation concealment methods. |
|  | 1.3 Did baseline differences between intervention groups suggest a problem with the randomization process? | N | The baseline was balanced. |
|  | **Risk of bias judgement** | **Some concerns** | - |
| **Bias due to deviations from intended interventions** | 2.1.Were participants aware of their assigned intervention during the trial? | NI | The author did not report blind method in detail. |
|  | 2.2.Were carers and people delivering the interventions aware of participants' assigned intervention during the trial? | NI | The author did not report blind method in detail. |
|  | 2.3. If Y/PY/NI to 2.1 or 2.2: Were there deviations from the intended intervention that arose because of the experimental context? | NI | No enough information. |
|  | 2.4 If Y/PY to 2.3: Were these deviations likely to have affected the outcome? | NA | - |
|  | 2.5. If Y/PY/NI to 2.4: Were these deviations from intended intervention balanced between groups? | NA | - |
|  | 2.6 Was an appropriate analysis used to estimate the effect of assignment to intervention? | NI | No enough information. |
|  | 2.7 If N/PN/NI to 2.6: Was there potential for a substantial impact (on the result) of the failure to analyse participants in the group to which they were randomized? | NI | No enough information. |
|  | **Risk of bias judgement** | **High** | - |
| **Bias due to missing outcome data** | 3.1 Were data for this outcome available for all, or nearly all, participants randomized? | N | The author reported six patients lost to follow-up but did not elaborate on the reasons. |
|  | 3.2 If N/PN/NI to 3.1: Is there evidence that result was not biased by missing outcome data? | N | No enough information. |
|  | 3.3 If N/PN to 3.2: Could missingness in the outcome depend on its true value? | NI | No enough information. |
|  | 3.4 If Y/PY/NI to 3.3: Is it likely that missingness in the outcome depended on its true value? | NI | No enough information. |
|  | **Risk of bias judgement** | **High** | - |
| **Bias in measurement of the outcome** | 4.1 Was the method of measuring the outcome inappropriate? | N | The method was appropriate. |
|  | 4.2 Could measurement or ascertainment of the outcome have differed between intervention groups? | N | There was no difference between groups. |
|  | 4.3 Were outcome assessors aware of the intervention received by study participants? | NI | The author did not report blind method in detail. |
|  | 4.4 If Y/PY/NI to 4.3: Could assessment of the outcome have been influenced by knowledge of intervention received? | NI | No enough information. |
|  | 4.5 If Y/PY/NI to 4.4: Is it likely that assessment of the outcome was influenced by knowledge of intervention received? | NI | No enough information. |
|  | **Risk of bias judgement** | **High** | - |
| **Bias in selection of the reported result** | 5.1 Were the data that produced this result analysed in accordance with a pre-specified analysis plan that was finalized before unblinded outcome data were available for analysis? | NI | The protocol can not be obtained. |
|  | 5.2 ... multiple eligible outcome measurements (e.g. scales, definitions, time points) within the outcome domain? | NI | The protocol can not be obtained. |
|  | 5.3 ... multiple eligible analyses of the data? | NI | The protocol can not be obtained. |
|  | **Risk of bias judgement** | **Some concerns** | - |
| **Overall bias** | **Risk of bias judgement** | **High** | - |

Fu CH 2021

| **Domain** | **Signalling question** | **Response** | **Comments** |
| --- | --- | --- | --- |
| **Bias arising from the randomization process** | 1.1 Was the allocation sequence random? | Y | The study used the random number table method to group patients. |
|  | 1.2 Was the allocation sequence concealed until participants were enrolled and assigned to interventions? | NI | The authors did not report specific allocation concealment methods. |
|  | 1.3 Did baseline differences between intervention groups suggest a problem with the randomization process? | N | The baseline was balanced. |
|  | **Risk of bias judgement** | **Some concerns** | - |
| **Bias due to deviations from intended interventions** | 2.1.Were participants aware of their assigned intervention during the trial? | NI | The author did not report blind method in detail. |
|  | 2.2.Were carers and people delivering the interventions aware of participants' assigned intervention during the trial? | NI | The author did not report blind method in detail. |
|  | 2.3. If Y/PY/NI to 2.1 or 2.2: Were there deviations from the intended intervention that arose because of the experimental context? | NI | No enough information. |
|  | 2.4 If Y/PY to 2.3: Were these deviations likely to have affected the outcome? | NA | - |
|  | 2.5. If Y/PY/NI to 2.4: Were these deviations from intended intervention balanced between groups? | NA | - |
|  | 2.6 Was an appropriate analysis used to estimate the effect of assignment to intervention? | NI | No enough information. |
|  | 2.7 If N/PN/NI to 2.6: Was there potential for a substantial impact (on the result) of the failure to analyse participants in the group to which they were randomized? | NI | No enough information. |
|  | **Risk of bias judgement** | **High** | - |
| **Bias due to missing outcome data** | 3.1 Were data for this outcome available for all, or nearly all, participants randomized? | Y | The author reported data for all subjects participating in randomization. |
|  | 3.2 If N/PN/NI to 3.1: Is there evidence that result was not biased by missing outcome data? | NA | - |
|  | 3.3 If N/PN to 3.2: Could missingness in the outcome depend on its true value? | NA | - |
|  | 3.4 If Y/PY/NI to 3.3: Is it likely that missingness in the outcome depended on its true value? | NA | - |
|  | **Risk of bias judgement** | **Low** | - |
| **Bias in measurement of the outcome** | 4.1 Was the method of measuring the outcome inappropriate? | N | The method was appropriate. |
|  | 4.2 Could measurement or ascertainment of the outcome have differed between intervention groups? | N | There was no difference between groups. |
|  | 4.3 Were outcome assessors aware of the intervention received by study participants? | NI | The author did not report blind method in detail. |
|  | 4.4 If Y/PY/NI to 4.3: Could assessment of the outcome have been influenced by knowledge of intervention received? | NI | No enough information. |
|  | 4.5 If Y/PY/NI to 4.4: Is it likely that assessment of the outcome was influenced by knowledge of intervention received? | NI | No enough information. |
|  | **Risk of bias judgement** | **High** | - |
| **Bias in selection of the reported result** | 5.1 Were the data that produced this result analysed in accordance with a pre-specified analysis plan that was finalized before unblinded outcome data were available for analysis? | NI | The protocol can not be obtained. |
|  | 5.2 ... multiple eligible outcome measurements (e.g. scales, definitions, time points) within the outcome domain? | NI | The protocol can not be obtained. |
|  | 5.3 ... multiple eligible analyses of the data? | NI | The protocol can not be obtained. |
|  | **Risk of bias judgement** | **Some concerns** | - |
| **Overall bias** | **Risk of bias judgement** | **High** | - |

Shen SX 2021

| **Domain** | **Signalling question** | **Response** | **Comments** |
| --- | --- | --- | --- |
| **Bias arising from the randomization process** | 1.1 Was the allocation sequence random? | Y | The study used the random number table method to group patients. |
|  | 1.2 Was the allocation sequence concealed until participants were enrolled and assigned to interventions? | NI | The authors did not report specific allocation concealment methods. |
|  | 1.3 Did baseline differences between intervention groups suggest a problem with the randomization process? | N | The baseline was balanced. |
|  | **Risk of bias judgement** | **Some concerns** | - |
| **Bias due to deviations from intended interventions** | 2.1.Were participants aware of their assigned intervention during the trial? | NI | The author did not report blind method in detail. |
|  | 2.2.Were carers and people delivering the interventions aware of participants' assigned intervention during the trial? | NI | The author did not report blind method in detail. |
|  | 2.3. If Y/PY/NI to 2.1 or 2.2: Were there deviations from the intended intervention that arose because of the experimental context? | NI | No enough information. |
|  | 2.4 If Y/PY to 2.3: Were these deviations likely to have affected the outcome? | NA | - |
|  | 2.5. If Y/PY/NI to 2.4: Were these deviations from intended intervention balanced between groups? | NA | - |
|  | 2.6 Was an appropriate analysis used to estimate the effect of assignment to intervention? | NI | No enough information. |
|  | 2.7 If N/PN/NI to 2.6: Was there potential for a substantial impact (on the result) of the failure to analyse participants in the group to which they were randomized? | NI | No enough information. |
|  | **Risk of bias judgement** | **High** | - |
| **Bias due to missing outcome data** | 3.1 Were data for this outcome available for all, or nearly all, participants randomized? | Y | The author reported data for all subjects participating in randomization. |
|  | 3.2 If N/PN/NI to 3.1: Is there evidence that result was not biased by missing outcome data? | NA | - |
|  | 3.3 If N/PN to 3.2: Could missingness in the outcome depend on its true value? | NA | - |
|  | 3.4 If Y/PY/NI to 3.3: Is it likely that missingness in the outcome depended on its true value? | NA | - |
|  | **Risk of bias judgement** | **Low** | - |
| **Bias in measurement of the outcome** | 4.1 Was the method of measuring the outcome inappropriate? | N | The method was appropriate. |
|  | 4.2 Could measurement or ascertainment of the outcome have differed between intervention groups? | N | There was no difference between groups. |
|  | 4.3 Were outcome assessors aware of the intervention received by study participants? | NI | The author did not report blind method in detail. |
|  | 4.4 If Y/PY/NI to 4.3: Could assessment of the outcome have been influenced by knowledge of intervention received? | NI | No enough information. |
|  | 4.5 If Y/PY/NI to 4.4: Is it likely that assessment of the outcome was influenced by knowledge of intervention received? | NI | No enough information. |
|  | **Risk of bias judgement** | **High** | - |
| **Bias in selection of the reported result** | 5.1 Were the data that produced this result analysed in accordance with a pre-specified analysis plan that was finalized before unblinded outcome data were available for analysis? | NI | The protocol can not be obtained. |
|  | 5.2 ... multiple eligible outcome measurements (e.g. scales, definitions, time points) within the outcome domain? | NI | The protocol can not be obtained. |
|  | 5.3 ... multiple eligible analyses of the data? | NI | The protocol can not be obtained. |
|  | **Risk of bias judgement** | **Some concerns** | - |
| **Overall bias** | **Risk of bias judgement** | **High** | - |

Song KY 2011

| **Domain** | **Signalling question** | **Response** | **Comments** |
| --- | --- | --- | --- |
| **Bias arising from the randomization process** | 1.1 Was the allocation sequence random? | NI | The authors did not report specific randomization methods. |
|  | 1.2 Was the allocation sequence concealed until participants were enrolled and assigned to interventions? | NI | The authors did not report specific allocation concealment methods. |
|  | 1.3 Did baseline differences between intervention groups suggest a problem with the randomization process? | N | The baseline was balanced. |
|  | **Risk of bias judgement** | **Some concerns** | - |
| **Bias due to deviations from intended interventions** | 2.1.Were participants aware of their assigned intervention during the trial? | NI | The author did not report blind method in detail. |
|  | 2.2.Were carers and people delivering the interventions aware of participants' assigned intervention during the trial? | NI | The author did not report blind method in detail. |
|  | 2.3. If Y/PY/NI to 2.1 or 2.2: Were there deviations from the intended intervention that arose because of the experimental context? | NI | No enough information. |
|  | 2.4 If Y/PY to 2.3: Were these deviations likely to have affected the outcome? | NA | - |
|  | 2.5. If Y/PY/NI to 2.4: Were these deviations from intended intervention balanced between groups? | NA | - |
|  | 2.6 Was an appropriate analysis used to estimate the effect of assignment to intervention? | NI | No enough information. |
|  | 2.7 If N/PN/NI to 2.6: Was there potential for a substantial impact (on the result) of the failure to analyse participants in the group to which they were randomized? | NI | No enough information. |
|  | **Risk of bias judgement** | **High** | - |
| **Bias due to missing outcome data** | 3.1 Were data for this outcome available for all, or nearly all, participants randomized? | Y | The author reported data for all subjects participating in randomization. |
|  | 3.2 If N/PN/NI to 3.1: Is there evidence that result was not biased by missing outcome data? | NA | - |
|  | 3.3 If N/PN to 3.2: Could missingness in the outcome depend on its true value? | NA | - |
|  | 3.4 If Y/PY/NI to 3.3: Is it likely that missingness in the outcome depended on its true value? | NA | - |
|  | **Risk of bias judgement** | **Low** | - |
| **Bias in measurement of the outcome** | 4.1 Was the method of measuring the outcome inappropriate? | N | The method was appropriate. |
|  | 4.2 Could measurement or ascertainment of the outcome have differed between intervention groups? | N | There was no difference between groups. |
|  | 4.3 Were outcome assessors aware of the intervention received by study participants? | NI | The author did not report blind method in detail. |
|  | 4.4 If Y/PY/NI to 4.3: Could assessment of the outcome have been influenced by knowledge of intervention received? | NI | No enough information. |
|  | 4.5 If Y/PY/NI to 4.4: Is it likely that assessment of the outcome was influenced by knowledge of intervention received? | NI | No enough information. |
|  | **Risk of bias judgement** | **High** | - |
| **Bias in selection of the reported result** | 5.1 Were the data that produced this result analysed in accordance with a pre-specified analysis plan that was finalized before unblinded outcome data were available for analysis? | NI | The protocol can not be obtained. |
|  | 5.2 ... multiple eligible outcome measurements (e.g. scales, definitions, time points) within the outcome domain? | NI | The protocol can not be obtained. |
|  | 5.3 ... multiple eligible analyses of the data? | NI | The protocol can not be obtained. |
|  | **Risk of bias judgement** | **Some concerns** | - |
| **Overall bias** | **Risk of bias judgement** | **High** | - |

Song Z 2021

| **Domain** | **Signalling question** | **Response** | **Comments** |
| --- | --- | --- | --- |
| **Bias arising from the randomization process** | 1.1 Was the allocation sequence random? | Y | The study used the random number table method to group patients. |
|  | 1.2 Was the allocation sequence concealed until participants were enrolled and assigned to interventions? | NI | The authors did not report specific allocation concealment methods. |
|  | 1.3 Did baseline differences between intervention groups suggest a problem with the randomization process? | N | The baseline was balanced. |
|  | **Risk of bias judgement** | **Some concerns** | - |
| **Bias due to deviations from intended interventions** | 2.1.Were participants aware of their assigned intervention during the trial? | NI | The author did not report blind method in detail. |
|  | 2.2.Were carers and people delivering the interventions aware of participants' assigned intervention during the trial? | NI | The author did not report blind method in detail. |
|  | 2.3. If Y/PY/NI to 2.1 or 2.2: Were there deviations from the intended intervention that arose because of the experimental context? | NI | No enough information. |
|  | 2.4 If Y/PY to 2.3: Were these deviations likely to have affected the outcome? | NA | - |
|  | 2.5. If Y/PY/NI to 2.4: Were these deviations from intended intervention balanced between groups? | NA | - |
|  | 2.6 Was an appropriate analysis used to estimate the effect of assignment to intervention? | NI | No enough information. |
|  | 2.7 If N/PN/NI to 2.6: Was there potential for a substantial impact (on the result) of the failure to analyse participants in the group to which they were randomized? | NI | No enough information. |
|  | **Risk of bias judgement** | **High** | - |
| **Bias due to missing outcome data** | 3.1 Were data for this outcome available for all, or nearly all, participants randomized? | Y | The author reported data for all subjects participating in randomization. |
|  | 3.2 If N/PN/NI to 3.1: Is there evidence that result was not biased by missing outcome data? | NA | - |
|  | 3.3 If N/PN to 3.2: Could missingness in the outcome depend on its true value? | NA | - |
|  | 3.4 If Y/PY/NI to 3.3: Is it likely that missingness in the outcome depended on its true value? | NA | - |
|  | **Risk of bias judgement** | **Low** | - |
| **Bias in measurement of the outcome** | 4.1 Was the method of measuring the outcome inappropriate? | N | The method was appropriate. |
|  | 4.2 Could measurement or ascertainment of the outcome have differed between intervention groups? | N | There was no difference between groups. |
|  | 4.3 Were outcome assessors aware of the intervention received by study participants? | NI | The author did not report blind method in detail. |
|  | 4.4 If Y/PY/NI to 4.3: Could assessment of the outcome have been influenced by knowledge of intervention received? | NI | No enough information. |
|  | 4.5 If Y/PY/NI to 4.4: Is it likely that assessment of the outcome was influenced by knowledge of intervention received? | NI | No enough information. |
|  | **Risk of bias judgement** | **High** | - |
| **Bias in selection of the reported result** | 5.1 Were the data that produced this result analysed in accordance with a pre-specified analysis plan that was finalized before unblinded outcome data were available for analysis? | NI | The protocol can not be obtained. |
|  | 5.2 ... multiple eligible outcome measurements (e.g. scales, definitions, time points) within the outcome domain? | NI | The protocol can not be obtained. |
|  | 5.3 ... multiple eligible analyses of the data? | NI | The protocol can not be obtained. |
|  | **Risk of bias judgement** | **Some concerns** | - |
| **Overall bias** | **Risk of bias judgement** | **High** | - |

Sun XY 2020

| **Domain** | **Signalling question** | **Response** | **Comments** |
| --- | --- | --- | --- |
| **Bias arising from the randomization process** | 1.1 Was the allocation sequence random? | Y | The study used the random number table method to group patients. |
|  | 1.2 Was the allocation sequence concealed until participants were enrolled and assigned to interventions? | NI | The authors did not report specific allocation concealment methods. |
|  | 1.3 Did baseline differences between intervention groups suggest a problem with the randomization process? | N | The baseline was balanced. |
|  | **Risk of bias judgement** | **Some concerns** | - |
| **Bias due to deviations from intended interventions** | 2.1.Were participants aware of their assigned intervention during the trial? | NI | The author did not report blind method in detail. |
|  | 2.2.Were carers and people delivering the interventions aware of participants' assigned intervention during the trial? | NI | The author did not report blind method in detail. |
|  | 2.3. If Y/PY/NI to 2.1 or 2.2: Were there deviations from the intended intervention that arose because of the experimental context? | NI | No enough information. |
|  | 2.4 If Y/PY to 2.3: Were these deviations likely to have affected the outcome? | NA | - |
|  | 2.5. If Y/PY/NI to 2.4: Were these deviations from intended intervention balanced between groups? | NA | - |
|  | 2.6 Was an appropriate analysis used to estimate the effect of assignment to intervention? | NI | No enough information. |
|  | 2.7 If N/PN/NI to 2.6: Was there potential for a substantial impact (on the result) of the failure to analyse participants in the group to which they were randomized? | NI | No enough information. |
|  | **Risk of bias judgement** | **High** | - |
| **Bias due to missing outcome data** | 3.1 Were data for this outcome available for all, or nearly all, participants randomized? | Y | The author reported data for all subjects participating in randomization. |
|  | 3.2 If N/PN/NI to 3.1: Is there evidence that result was not biased by missing outcome data? | NA | - |
|  | 3.3 If N/PN to 3.2: Could missingness in the outcome depend on its true value? | NA | - |
|  | 3.4 If Y/PY/NI to 3.3: Is it likely that missingness in the outcome depended on its true value? | NA | - |
|  | **Risk of bias judgement** | **Low** | - |
| **Bias in measurement of the outcome** | 4.1 Was the method of measuring the outcome inappropriate? | N | The method was appropriate. |
|  | 4.2 Could measurement or ascertainment of the outcome have differed between intervention groups? | N | There was no difference between groups. |
|  | 4.3 Were outcome assessors aware of the intervention received by study participants? | NI | The author did not report blind method in detail. |
|  | 4.4 If Y/PY/NI to 4.3: Could assessment of the outcome have been influenced by knowledge of intervention received? | NI | No enough information. |
|  | 4.5 If Y/PY/NI to 4.4: Is it likely that assessment of the outcome was influenced by knowledge of intervention received? | NI | No enough information. |
|  | **Risk of bias judgement** | **High** | - |
| **Bias in selection of the reported result** | 5.1 Were the data that produced this result analysed in accordance with a pre-specified analysis plan that was finalized before unblinded outcome data were available for analysis? | NI | The protocol can not be obtained. |
|  | 5.2 ... multiple eligible outcome measurements (e.g. scales, definitions, time points) within the outcome domain? | NI | The protocol can not be obtained. |
|  | 5.3 ... multiple eligible analyses of the data? | NI | The protocol can not be obtained. |
|  | **Risk of bias judgement** | **Some concerns** | - |
| **Overall bias** | **Risk of bias judgement** | **High** | - |

Wang HZ 2015

| **Domain** | **Signalling question** | **Response** | **Comments** |
| --- | --- | --- | --- |
| **Bias arising from the randomization process** | 1.1 Was the allocation sequence random? | NI | The authors did not report specific randomization methods. |
|  | 1.2 Was the allocation sequence concealed until participants were enrolled and assigned to interventions? | NI | The authors did not report specific allocation concealment methods. |
|  | 1.3 Did baseline differences between intervention groups suggest a problem with the randomization process? | N | The baseline was balanced. |
|  | **Risk of bias judgement** | **Some concerns** | - |
| **Bias due to deviations from intended interventions** | 2.1.Were participants aware of their assigned intervention during the trial? | NI | The author did not report blind method in detail. |
|  | 2.2.Were carers and people delivering the interventions aware of participants' assigned intervention during the trial? | NI | The author did not report blind method in detail. |
|  | 2.3. If Y/PY/NI to 2.1 or 2.2: Were there deviations from the intended intervention that arose because of the experimental context? | NI | No enough information. |
|  | 2.4 If Y/PY to 2.3: Were these deviations likely to have affected the outcome? | NA | - |
|  | 2.5. If Y/PY/NI to 2.4: Were these deviations from intended intervention balanced between groups? | NA | - |
|  | 2.6 Was an appropriate analysis used to estimate the effect of assignment to intervention? | NI | No enough information. |
|  | 2.7 If N/PN/NI to 2.6: Was there potential for a substantial impact (on the result) of the failure to analyse participants in the group to which they were randomized? | NI | No enough information. |
|  | **Risk of bias judgement** | **High** | - |
| **Bias due to missing outcome data** | 3.1 Were data for this outcome available for all, or nearly all, participants randomized? | Y | The author reported data for all subjects participating in randomization. |
|  | 3.2 If N/PN/NI to 3.1: Is there evidence that result was not biased by missing outcome data? | NA | - |
|  | 3.3 If N/PN to 3.2: Could missingness in the outcome depend on its true value? | NA | - |
|  | 3.4 If Y/PY/NI to 3.3: Is it likely that missingness in the outcome depended on its true value? | NA | - |
|  | **Risk of bias judgement** | **Low** | - |
| **Bias in measurement of the outcome** | 4.1 Was the method of measuring the outcome inappropriate? | N | The method was appropriate. |
|  | 4.2 Could measurement or ascertainment of the outcome have differed between intervention groups? | N | There was no difference between groups. |
|  | 4.3 Were outcome assessors aware of the intervention received by study participants? | NI | The author did not report blind method in detail. |
|  | 4.4 If Y/PY/NI to 4.3: Could assessment of the outcome have been influenced by knowledge of intervention received? | NI | No enough information. |
|  | 4.5 If Y/PY/NI to 4.4: Is it likely that assessment of the outcome was influenced by knowledge of intervention received? | NI | No enough information. |
|  | **Risk of bias judgement** | **High** | - |
| **Bias in selection of the reported result** | 5.1 Were the data that produced this result analysed in accordance with a pre-specified analysis plan that was finalized before unblinded outcome data were available for analysis? | NI | The protocol can not be obtained. |
|  | 5.2 ... multiple eligible outcome measurements (e.g. scales, definitions, time points) within the outcome domain? | NI | The protocol can not be obtained. |
|  | 5.3 ... multiple eligible analyses of the data? | NI | The protocol can not be obtained. |
|  | **Risk of bias judgement** | **Some concerns** | - |
| **Overall bias** | **Risk of bias judgement** | **High** | - |

Wu CY 2019

| **Domain** | **Signalling question** | **Response** | **Comments** |
| --- | --- | --- | --- |
| **Bias arising from the randomization process** | 1.1 Was the allocation sequence random? | NI | The authors did not report specific randomization methods. |
|  | 1.2 Was the allocation sequence concealed until participants were enrolled and assigned to interventions? | NI | The authors did not report specific allocation concealment methods. |
|  | 1.3 Did baseline differences between intervention groups suggest a problem with the randomization process? | N | The baseline was balanced. |
|  | **Risk of bias judgement** | **Some concerns** | - |
| **Bias due to deviations from intended interventions** | 2.1.Were participants aware of their assigned intervention during the trial? | NI | The author did not report blind method in detail. |
|  | 2.2.Were carers and people delivering the interventions aware of participants' assigned intervention during the trial? | NI | The author did not report blind method in detail. |
|  | 2.3. If Y/PY/NI to 2.1 or 2.2: Were there deviations from the intended intervention that arose because of the experimental context? | NI | No enough information. |
|  | 2.4 If Y/PY to 2.3: Were these deviations likely to have affected the outcome? | NA | - |
|  | 2.5. If Y/PY/NI to 2.4: Were these deviations from intended intervention balanced between groups? | NA | - |
|  | 2.6 Was an appropriate analysis used to estimate the effect of assignment to intervention? | NI | No enough information. |
|  | 2.7 If N/PN/NI to 2.6: Was there potential for a substantial impact (on the result) of the failure to analyse participants in the group to which they were randomized? | NI | No enough information. |
|  | **Risk of bias judgement** | **High** | - |
| **Bias due to missing outcome data** | 3.1 Were data for this outcome available for all, or nearly all, participants randomized? | Y | The author reported data for all subjects participating in randomization. |
|  | 3.2 If N/PN/NI to 3.1: Is there evidence that result was not biased by missing outcome data? | NA | - |
|  | 3.3 If N/PN to 3.2: Could missingness in the outcome depend on its true value? | NA | - |
|  | 3.4 If Y/PY/NI to 3.3: Is it likely that missingness in the outcome depended on its true value? | NA | - |
|  | **Risk of bias judgement** | **Low** | - |
| **Bias in measurement of the outcome** | 4.1 Was the method of measuring the outcome inappropriate? | N | The method was appropriate. |
|  | 4.2 Could measurement or ascertainment of the outcome have differed between intervention groups? | N | There was no difference between groups. |
|  | 4.3 Were outcome assessors aware of the intervention received by study participants? | NI | The author did not report blind method in detail. |
|  | 4.4 If Y/PY/NI to 4.3: Could assessment of the outcome have been influenced by knowledge of intervention received? | NI | No enough information. |
|  | 4.5 If Y/PY/NI to 4.4: Is it likely that assessment of the outcome was influenced by knowledge of intervention received? | NI | No enough information. |
|  | **Risk of bias judgement** | **High** | - |
| **Bias in selection of the reported result** | 5.1 Were the data that produced this result analysed in accordance with a pre-specified analysis plan that was finalized before unblinded outcome data were available for analysis? | NI | The protocol can not be obtained. |
|  | 5.2 ... multiple eligible outcome measurements (e.g. scales, definitions, time points) within the outcome domain? | NI | The protocol can not be obtained. |
|  | 5.3 ... multiple eligible analyses of the data? | NI | The protocol can not be obtained. |
|  | **Risk of bias judgement** | **Some concerns** | - |
| **Overall bias** | **Risk of bias judgement** | **High** | - |

Yan XY 2021

| **Domain** | **Signalling question** | **Response** | **Comments** |
| --- | --- | --- | --- |
| **Bias arising from the randomization process** | 1.1 Was the allocation sequence random? | NI | The authors did not report specific randomization methods. |
|  | 1.2 Was the allocation sequence concealed until participants were enrolled and assigned to interventions? | NI | The authors did not report specific allocation concealment methods. |
|  | 1.3 Did baseline differences between intervention groups suggest a problem with the randomization process? | N | The baseline was balanced. |
|  | **Risk of bias judgement** | **Some concerns** | - |
| **Bias due to deviations from intended interventions** | 2.1.Were participants aware of their assigned intervention during the trial? | NI | The author did not report blind method in detail. |
|  | 2.2.Were carers and people delivering the interventions aware of participants' assigned intervention during the trial? | NI | The author did not report blind method in detail. |
|  | 2.3. If Y/PY/NI to 2.1 or 2.2: Were there deviations from the intended intervention that arose because of the experimental context? | NI | No enough information. |
|  | 2.4 If Y/PY to 2.3: Were these deviations likely to have affected the outcome? | NA | - |
|  | 2.5. If Y/PY/NI to 2.4: Were these deviations from intended intervention balanced between groups? | NA | - |
|  | 2.6 Was an appropriate analysis used to estimate the effect of assignment to intervention? | NI | No enough information. |
|  | 2.7 If N/PN/NI to 2.6: Was there potential for a substantial impact (on the result) of the failure to analyse participants in the group to which they were randomized? | NI | No enough information. |
|  | **Risk of bias judgement** | **High** | - |
| **Bias due to missing outcome data** | 3.1 Were data for this outcome available for all, or nearly all, participants randomized? | Y | The author reported data for all subjects participating in randomization. |
|  | 3.2 If N/PN/NI to 3.1: Is there evidence that result was not biased by missing outcome data? | NA | - |
|  | 3.3 If N/PN to 3.2: Could missingness in the outcome depend on its true value? | NA | - |
|  | 3.4 If Y/PY/NI to 3.3: Is it likely that missingness in the outcome depended on its true value? | NA | - |
|  | **Risk of bias judgement** | **Low** | - |
| **Bias in measurement of the outcome** | 4.1 Was the method of measuring the outcome inappropriate? | N | The method was appropriate. |
|  | 4.2 Could measurement or ascertainment of the outcome have differed between intervention groups? | N | There was no difference between groups. |
|  | 4.3 Were outcome assessors aware of the intervention received by study participants? | NI | The author did not report blind method in detail. |
|  | 4.4 If Y/PY/NI to 4.3: Could assessment of the outcome have been influenced by knowledge of intervention received? | NI | No enough information. |
|  | 4.5 If Y/PY/NI to 4.4: Is it likely that assessment of the outcome was influenced by knowledge of intervention received? | NI | No enough information. |
|  | **Risk of bias judgement** | **High** | - |
| **Bias in selection of the reported result** | 5.1 Were the data that produced this result analysed in accordance with a pre-specified analysis plan that was finalized before unblinded outcome data were available for analysis? | NI | The protocol can not be obtained. |
|  | 5.2 ... multiple eligible outcome measurements (e.g. scales, definitions, time points) within the outcome domain? | NI | The protocol can not be obtained. |
|  | 5.3 ... multiple eligible analyses of the data? | NI | The protocol can not be obtained. |
|  | **Risk of bias judgement** | **Some concerns** | - |
| **Overall bias** | **Risk of bias judgement** | **High** | - |

Zhang HF 2020

| **Domain** | **Signalling question** | **Response** | **Comments** |
| --- | --- | --- | --- |
| **Bias arising from the randomization process** | 1.1 Was the allocation sequence random? | NI | The authors did not report specific randomization methods. |
|  | 1.2 Was the allocation sequence concealed until participants were enrolled and assigned to interventions? | NI | The authors did not report specific allocation concealment methods. |
|  | 1.3 Did baseline differences between intervention groups suggest a problem with the randomization process? | N | The baseline was balanced. |
|  | **Risk of bias judgement** | **Some concerns** | - |
| **Bias due to deviations from intended interventions** | 2.1.Were participants aware of their assigned intervention during the trial? | NI | The author did not report blind method in detail. |
|  | 2.2.Were carers and people delivering the interventions aware of participants' assigned intervention during the trial? | NI | The author did not report blind method in detail. |
|  | 2.3. If Y/PY/NI to 2.1 or 2.2: Were there deviations from the intended intervention that arose because of the experimental context? | NI | No enough information. |
|  | 2.4 If Y/PY to 2.3: Were these deviations likely to have affected the outcome? | NA | - |
|  | 2.5. If Y/PY/NI to 2.4: Were these deviations from intended intervention balanced between groups? | NA | - |
|  | 2.6 Was an appropriate analysis used to estimate the effect of assignment to intervention? | NI | No enough information. |
|  | 2.7 If N/PN/NI to 2.6: Was there potential for a substantial impact (on the result) of the failure to analyse participants in the group to which they were randomized? | NI | No enough information. |
|  | **Risk of bias judgement** | **High** | - |
| **Bias due to missing outcome data** | 3.1 Were data for this outcome available for all, or nearly all, participants randomized? | Y | The author reported data for all subjects participating in randomization. |
|  | 3.2 If N/PN/NI to 3.1: Is there evidence that result was not biased by missing outcome data? | NA | - |
|  | 3.3 If N/PN to 3.2: Could missingness in the outcome depend on its true value? | NA | - |
|  | 3.4 If Y/PY/NI to 3.3: Is it likely that missingness in the outcome depended on its true value? | NA | - |
|  | **Risk of bias judgement** | **Low** | - |
| **Bias in measurement of the outcome** | 4.1 Was the method of measuring the outcome inappropriate? | N | The method was appropriate. |
|  | 4.2 Could measurement or ascertainment of the outcome have differed between intervention groups? | N | There was no difference between groups. |
|  | 4.3 Were outcome assessors aware of the intervention received by study participants? | NI | The author did not report blind method in detail. |
|  | 4.4 If Y/PY/NI to 4.3: Could assessment of the outcome have been influenced by knowledge of intervention received? | NI | No enough information. |
|  | 4.5 If Y/PY/NI to 4.4: Is it likely that assessment of the outcome was influenced by knowledge of intervention received? | NI | No enough information. |
|  | **Risk of bias judgement** | **High** | - |
| **Bias in selection of the reported result** | 5.1 Were the data that produced this result analysed in accordance with a pre-specified analysis plan that was finalized before unblinded outcome data were available for analysis? | NI | The protocol can not be obtained. |
|  | 5.2 ... multiple eligible outcome measurements (e.g. scales, definitions, time points) within the outcome domain? | NI | The protocol can not be obtained. |
|  | 5.3 ... multiple eligible analyses of the data? | NI | The protocol can not be obtained. |
|  | **Risk of bias judgement** | **Some concerns** | - |
| **Overall bias** | **Risk of bias judgement** | **High** | - |

Zhang LW 2019

| **Domain** | **Signalling question** | **Response** | **Comments** |
| --- | --- | --- | --- |
| **Bias arising from the randomization process** | 1.1 Was the allocation sequence random? | NI | The authors did not report specific randomization methods. |
|  | 1.2 Was the allocation sequence concealed until participants were enrolled and assigned to interventions? | NI | The authors did not report specific allocation concealment methods. |
|  | 1.3 Did baseline differences between intervention groups suggest a problem with the randomization process? | N | The baseline was balanced. |
|  | **Risk of bias judgement** | **Some concerns** | - |
| **Bias due to deviations from intended interventions** | 2.1.Were participants aware of their assigned intervention during the trial? | NI | The author did not report blind method in detail. |
|  | 2.2.Were carers and people delivering the interventions aware of participants' assigned intervention during the trial? | NI | The author did not report blind method in detail. |
|  | 2.3. If Y/PY/NI to 2.1 or 2.2: Were there deviations from the intended intervention that arose because of the experimental context? | NI | No enough information. |
|  | 2.4 If Y/PY to 2.3: Were these deviations likely to have affected the outcome? | NA | - |
|  | 2.5. If Y/PY/NI to 2.4: Were these deviations from intended intervention balanced between groups? | NA | - |
|  | 2.6 Was an appropriate analysis used to estimate the effect of assignment to intervention? | NI | No enough information. |
|  | 2.7 If N/PN/NI to 2.6: Was there potential for a substantial impact (on the result) of the failure to analyse participants in the group to which they were randomized? | NI | No enough information. |
|  | **Risk of bias judgement** | **High** | - |
| **Bias due to missing outcome data** | 3.1 Were data for this outcome available for all, or nearly all, participants randomized? | Y | The author reported data for all subjects participating in randomization. |
|  | 3.2 If N/PN/NI to 3.1: Is there evidence that result was not biased by missing outcome data? | NA | - |
|  | 3.3 If N/PN to 3.2: Could missingness in the outcome depend on its true value? | NA | - |
|  | 3.4 If Y/PY/NI to 3.3: Is it likely that missingness in the outcome depended on its true value? | NA | - |
|  | **Risk of bias judgement** | **Low** | - |
| **Bias in measurement of the outcome** | 4.1 Was the method of measuring the outcome inappropriate? | N | The method was appropriate. |
|  | 4.2 Could measurement or ascertainment of the outcome have differed between intervention groups? | N | There was no difference between groups. |
|  | 4.3 Were outcome assessors aware of the intervention received by study participants? | NI | The author did not report blind method in detail. |
|  | 4.4 If Y/PY/NI to 4.3: Could assessment of the outcome have been influenced by knowledge of intervention received? | NI | No enough information. |
|  | 4.5 If Y/PY/NI to 4.4: Is it likely that assessment of the outcome was influenced by knowledge of intervention received? | NI | No enough information. |
|  | **Risk of bias judgement** | **High** | - |
| **Bias in selection of the reported result** | 5.1 Were the data that produced this result analysed in accordance with a pre-specified analysis plan that was finalized before unblinded outcome data were available for analysis? | NI | The protocol can not be obtained. |
|  | 5.2 ... multiple eligible outcome measurements (e.g. scales, definitions, time points) within the outcome domain? | NI | The protocol can not be obtained. |
|  | 5.3 ... multiple eligible analyses of the data? | NI | The protocol can not be obtained. |
|  | **Risk of bias judgement** | **Some concerns** | - |
| **Overall bias** | **Risk of bias judgement** | **High** | - |
